# Engineering *Corynebacterium glutamicum* for violacein hyper production

**DOI:** 10.1186/s12934-016-0545-0

**Published:** 2016-08-24

**Authors:** Hongnian Sun, Dongdong Zhao, Bin Xiong, Chunzhi Zhang, Changhao Bi

**Affiliations:** 1 School of Biological Engineering, Dalian Polytechnic University, Dalian, 116034 People’s Republic of China; 2 Tianjin Institute of Industrial Biotechnology, Chinese Academy of Sciences, Tianjin, 300308 People’s Republic of China

**Keywords:** *Corynebacterium glutamicum*, Metabolic engineering, Violacein, l-Tryptophan

## Abstract

**Background:**

*Corynebacterium glutamicum* was used as a metabolic engineering chassis for production of crude violacein (mixture of violacein and deoxyviolacein) due to *Corynebacterium*’s GRAS status and advantages in tryptophan fermentation. The violacein is a commercially potential compound with various applications derived from l-tryptophan.

**Results:**

*Corynebacterium glutamicum* ATCC 21850 that could produce 162.98 mg L^−1^ tryptophan was employed as a novel host for metabolic engineering chassis. Heterologous *vio* operon from *Chromobacterium violaceum* was over-expressed in ATCC 21850 strain with constitutive promoter to have obtained 532 mg L^−1^ violacein. Considering toxicity of violacein, *vio* operon was expressed with inducible promoter and 629 mg L^−1^ violacein was obtained in batch culture. Due to the economical coding nature of *vio* operon, the compressed RBS of *vio* genes were replaced with complete strong *C. glutamicum* ones. And extended expression units were assembled to form a synthetic operon. With this strategy, 1116 mg L^−1^ violacein in batch culture was achieved. Fermentation process was then optimized by studying induction time, induction concentration, culture composition and fermentation temperature. as a result, a titer of 5436 mg L^−1^ and a productivity of 47 mg L^−1^ h^−1^ were achieved in 3 L bioreactor.

**Conclusions:**

With metabolic engineering and fermentation optimization practice, *C. glutamicum* 21850 (pEC-C-vio1) was able to produce violacein with both titer and productivity at the highest level ever reported. Due to advantages of mature *C. glutamicum* fermentation industry, this work has built basis for commercial production of violacein.

**Electronic supplementary material:**

The online version of this article (doi:10.1186/s12934-016-0545-0) contains supplementary material, which is available to authorized users.

## Background

Violacein is a natural indolocarbazole compound formed by condensation of two molecules of tryptophan [1], which was a potential novel pharmaceutical reagent due to its extensive biological properties such as antitumoral [2], antibacterial [3], antiviral [4], and antioxidant [5] activities [6]. Violacein biosynthesis begins with l-tryptophan and is catalyzed by enzymes VioA, B, E, D, and C successively, which were encoded by *vioABCDE* operon [7]. Several Gram-negative bacteria were reported to produce violacein as their secondary metabolites including *Chromobacterium violaceum, Janthinobacterium lividum, Alteromonas luteoviolacea, Pseudoalteromonas luteoviolacea*, *Duganella* sp. *B2,* etc. [6]. Some of the natural producers were studied to improve their violacein production, such as *C. violaceum* and *Duganella* sp*. B2* [8, 9]. However, fermentation by natural producers were limited for low productivity, bacterial pathogenicity, and difficulties in large scale culture [10, 11]. Due to these problems, recent research has been focused on metabolic engineering of l-tryptophan over-producing *Escherichia coli* strain for violacein production [10, 12]. Compared to *E. coli, C. glutamicum* is generally recognized as safes (GRAS) and exhibits numerous advantages as a microbial cell factory. *C. glutamicum* strains dominate industrial scale fermentation process to produce amino acids and other compounds for health, cosmetic, food and animal feed [13]. *C. glutamicum* strain ATCC 21850 is an established industrial l-tryptophan hyperproducer, which made it an attractive platform since l-tryptophan is direct precursor for the production of crude violacein. In this work, *C. glutamicum* strains including wild type ATCC 13032 and l-tryptophan producing were engineered as novel chassis for violacein production [14]. Various metabolic engineering and fermentation engineering strategies were applied to *C. glutamicum* ATCC 21850, and the reported highest violacein titer and productivity were achieved in this work.

## Methods

### Strains and media

Bacterial strains used in this study are listed in Table 1. pEC-XK99E [15], a *C. glutamicum/E. coli* shuttle expression vector, was used as plasmid backbone to construct function plasmids. *E. coli* DH5α was used for plasmid construction and cultured in Luria–Bertani (LB) broth at 37 °C. ATCC 13032 and ATCC 21850 were used as the host strains. *C. glutamicum* was cultivated at 30 °C in LBHIS medium [16]. Kanamycin at a final concentration of 50 or 25 μg mL^−1^ was added into the medium for cultivation of *E. coli* or *C. glutamicum* when needed.

**Table 1:** Strains and plasmids used and constructed in this study

Strains/plasmids	Relevant characteristics	Sources
*Strains*		
*E. coli* DH5α	F^−^ *endA1thi*-*1 recA1 relA1 gyrA96deoRΦ*80*dlac*Δ(*lac*Z) M15 Δ(*lacZYA*-*argF*)*U169hsdR17*(r_K_^−^, m_K_^+^) λ^–^ *supE44 phoA*	Invitrogen
ATCC 13032	*C. glutamicum* wild-type	ATCC
ATCC 21850	4-MT^r^ 5-MT^r^ 6-FT^r^ 4-Ap^r^ 4-FP^r^ TyrHx^r^ Phe^−^ Tyr^−^, tryptophan hyperproducer	ATCC
*Plasmids*		
pEC-XK99E	*C. glutamicum/E. coli* shuttle expression vector, Ptrc, lacIq, Kanr	Add gene
pEC-vioABCDE	derived from pEC-XK99E, constitutive expression of *C. violaceum vio* operon	This study
pEC-J-vio-1	pEC-XK99E derivative containing *vio* operon from *J. lividum*, expressed under control of inducible promoter Ptrc	This study
pEC-J-vio-2	pEC-XK99E derivative containing synthetic *J. lividum vio* operon with each gene containing complete *C. glutamicum* RBS sequence	This study
pEC-C-vio-1	pEC-XK99E derivative containing synthetic *C. violaceum vio* operon with each gene containing complete *C. glutamicum* RBS sequence	This study
pEC-C-vio-2	pEC-XK99E derivative containing synthetic *C. violaceum vio* operon with each gene containing complete *C. glutamicum* RBS sequence, which gene order changed to *vioB, vioA, vioE, vioC, vioD*	This study

In fermentative production of violacein with recombinant *C. glutamicum*, the seeding medium consisted of 50 g L^−1^ glucose, 10 g L^−1^ yeast extract, 30 g L^−1^ corn steep liquor, 4 g L^−1^ (NH_4_)_2_SO_4_, 0.25 g L^−1^ MgSO_4_·7H_2_O, 1 g L^−1^ KH_2_PO_4_, 1 g L^−1^ K_2_HPO_4_, 0.5 g L^−1^ Na_2_HPO_4_, 10 mg L^−1^ FeSO_4_·7H_2_O and 10 mg L^−1^ MnSO_4_·H_2_O, with pH adjusted to 7.2. The fermentation medium consisted of 60 g L^−1^ glucose, 50 g L^−1^ corn steep liquor, 10 g L^−1^ (NH_4_)_2_SO_4_, 0.5 g L^−1^ MgSO_4_·7H_2_O, 1 g L^−1^ KH_2_PO_4_, 1 g L^−1^ K_2_HPO_4_, 1 g L^−1^ Na_2_HPO_4_, 10 mg L^−1^ FeSO_4_·7H_2_O, 10 mg L^−1^ MnSO_4_·H_2_O, 30 mg L^−1^ phenylalanine, 30 mg L^−1^ tyrosine and 10 g L^−1^ CaCO_2_, with pH adjusted to 7.2. Glucose solution was sterilized separately.

### Culture conditions for cell growth and violacein production

For violacein production, a single colony from LBHIS plates was inoculated into a 100 mL flask containing 5 mL BHIS media, and cultured at 30 °C and 200 rpm on a rotary shake for 48 h. Then 1 mL of the culture was inoculated into 25 mL of the seed medium in a 250 mL rotary flask and cultivated at 30 °C and 200 rpm for 24 h.

A two-phase process was carried out for violacein production. Firstly, 1 mL of the seed culture was inoculated into 25 mL fermentation medium in a 500 mL rotary flask and cultivated at 30 °C 200 rpm for 12 h, then temperature was dropped from 30 to 20 °C to cultivate for 72 h. Induction was performed with addition of 0.5 mM isopropyl-β-d-thiogalactoside (IPTG) for strains with inducible expression promoters. Fermentation optimization experiments were carried out with various parameters, which might be different from this general condition, and was specified in the text and figure legends.

Fed-batch fermentations were performed in a 3 L fermenter (Bioflow 115, New Brunswick Scientific, USA) with a working volume of 1.5 L, whose fermentation conditions was similar to optimized batch cultures except that temperature of the second phase fermentation was shifted to 20 °C after 18 h. The dissolved oxygen level of the culture was controlled at about 30 % and the pH was maintained at 7.0 by automatic addition of 25 % NH_4_OH.

### Construction of plasmids

All plasmids were constructed by Golden-gate DNA assembly method [17], and amplified in *E. coli* DH5α. Then electrotransformed into *C. glutamicum* ATCC 13032 or ATCC 21850 as described previously [18].

For construction of pEC-vioABCDE, the *vio* operon was PCR amplified from *C. violaceum* genomic DNA with primers C-vio-F/C-vio-R. Assembly fragments pEC-ΔlacIq-P1 and pEC-ΔlacIq-P2 were amplified from pEC-XK99E with primers pEC-C1-F/pEC-C1-R and pEC-C2-F/pEC-C2-R respectively.

For construction of plasmid pEC-J-vio1, the *vio* operon was PCR amplified from the chromosomal DNA of *J. lividum* with primes J-vio-F and J-vio-R. Backbone of pEC-XK99E was divided into two segments to be amplified using primer pairs J-pec1-F/J-pec1-R and J-pec2-F/J-pec2-R respectively. For construction of plasmid pEC-J-vio2, the biosynthetic genes of *vioA, vioB, vioC, vioD, vioE* from *J. lividum* were PCR amplified from the chromosomal DNA with primers J-vioA-F/J-vioA-R, J-vioB-F/J-vioB-R, J-vioC-F/J-vioC-R, J-vioD-F/J-vioD-R, J-vioE-F/J-vioE-R respectively. The backbone was cloned from pEC-J-vio1 with primers J-pec1-F/J-pec2-R.

For construction of pEC-C-vio1, the *vio* operon was PCR amplified from pKMV-vioA, pKMV-vioB, pKMV-vioC, pKMV-vioD, pKMV-vioE with primers C-vioA-F/C-vioA-R, C-vioB-F/C-vioB-R, C-vioC-F/C-vioC-R, C-vioD-F/C-vioD-R, C-vioE-F/C-vioE-R respectively. Backbone was cloned from pEC-J-vio1 with primers C-vio-F/C-vio-R.

For construction of pEC-C-vio2, *vioB, vioA, vioE, vioC, vioD* were PCR amplified from pKMV-vioB, pKMV-vioA, pKMV-vioE, pKMV-vioC, pKMV-vioD with primers 2C-vioB-F/2C-vioB-R, 2C-vioA-F/2C-vioA-R, 2C-vioE-F/2C-vioE-R, 2C-vioC-F/2C-vioC-R, 2C-vioD-F/2C-vioD-R respectively. The backbone was amplified from pEC-J-vio1 with primers 2C-pec-F/2C-pec-R.

### Analytical method

500 μL samples were obtained from fermentation culture to determine violacein titer as reported previously [9, 12]. The correlation of Absorbance at 570 nm and concentration of crude violacein was determined as illustrated in (Additional file 1: Figure S1A), which was the standard for calculation. Detection of l-tryptophan in fermentation culture was carried by the approach as described recently [9, 12]. The correlation of Absorbance at 600 nm and concentration of l-tryptophan was determined as illustrated in (Additional file 1: Figure S1B), which was the standard for calculation. Glucose was determined using a biosensor analyzer (SBA-40B, Institute of Biology, Shandong Province Academy of Sciences, China). Fermentation broth was centrifuged and diluted 1:100 with 1 N HCl solutions to determine the OD_600_. Experiments were performed to find that OD_600_ of untreated samples were same as samples after violacein extracted with ethanol. The biomass of one unit OD_600_ was determined to equal around 0.3 g DCW L^−1^ (Additional file 1: Figure S1C), which was similar to previously reported value [19].

## Results and discussion

### *Corynebacterium glutamicum* was engineered to produce violacein

**Fig. 1:**
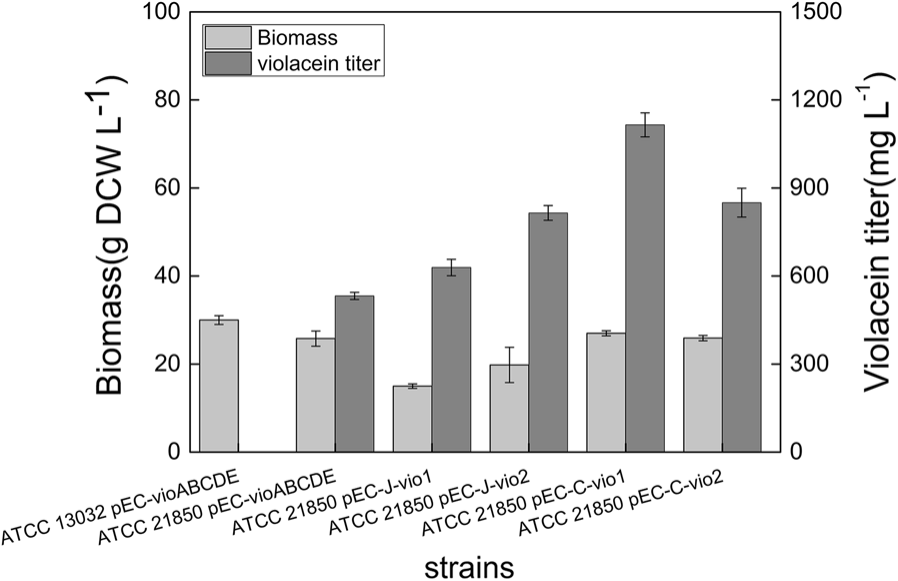
Batch fermentation results of engineered violacein prodcuing *C. glutamicum* strains. experiments were carried out in triplicate

*Corynebacterium glutamicum* was employed as metabolic engineering chassis for production of violacein. Plasmid pEC-XK99E-ΔlacIq was constructed with constitutively expressed vioABCDE operon from and transformed into the model strain *C. glutamicum* 13032. 13032 (pEC-vioABCDE) strain grew to a high cell density but produced no violacein in fermentation medium as illustrated in Fig. 1, although low titer of violacein was obtained in the rich medium LBHIS (Additional file 1: Figure S2). Then the tryptophan producer ATCC 21850 was used as chassis for engineering, which was able to accumulated 163 mg L^−1^l-tryptophan at 30 °C in 72 h. The resulting strain 21850 (pEC-vioABCDE) was successfully engineered and produced 532 mg L^−1^ violacein, higher than its tryptophan titer. This suggested that the violacein producing pathway was successfully expressed and functioned efficiently in ATCC 21850 strain. Probably due to better accumulation and excretion mechanism, violacein synthesis process even pulled more carbon flux to form tryptophan and subsequent violacein. This result proved tryptophan producing *C. glutamicum* a suitable host for production of violacein (Fig. 1). However, since violacein was reported to be an antibacterial reagent, the constitutive production might cause burden on cell, especially in the initial phase of growth. Thus, expression and regulation of *vio* genes should be further optimized for improved cell growth and violacein production.

### Metabolic engineering strategies were applied for improved violacein production

To avoid growth defect in initial growth phase, the *vio* operon vioABCDE, from *J. lividum* was constructed under regulation of Ptrc promoter to form plasmid pEC-J-vio1. When this plasmid was expressed in ATCC 21850, 629 mg L^−1^ violacein was produced when IPTG was added at 12 h after inoculation of seed culture (Fig. 1). This result supported our hypothesis that constitutive production of violacein affect its production.

**Fig. 2:**
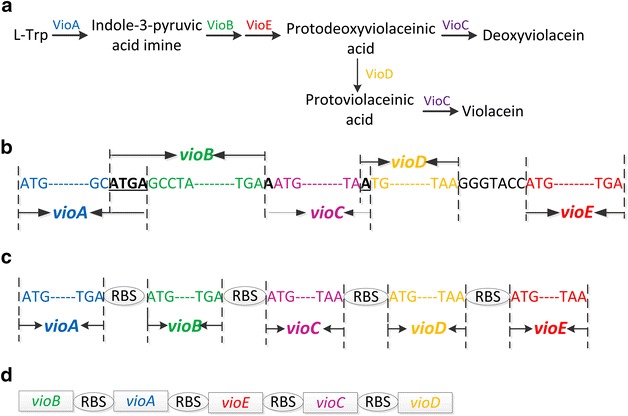
Illustration of engineering strategies with the *vio* operon. **a** Violacein synthetic pathway diagram. **b** Original form of *J. lividum vio* operon with compressed DNA sequence. **c** Synthetic *J. lividum vio* operon with extended complete RBS for each genes. **d** *vio* operon with altered gene order

The *vio* operon was found to have overlapping genes, which is common in rapidly evolving genomes with high mutation rates such as viruses and bacteria [20]. The main biological function of the overlapped genes was suggested be regulation of gene expression through translational coupling of functionally related polypeptides, and compression of maximum amount of information into shorter DNA sequences (Fig. 2). We hypothesized that such operon structure hampered its heterologous expression, and introduction of complete RBS sequences of the host bacteria to each gene might improve their expression. To test this hypothesis, *vio* genes from operon of *J. lividum* were individually cloned. Their RBSs were replaced with strong *C. glutamicum* RBSs (GAAAGGAGGTTTGGACA) individually [21], and assembled to form a novel synthetic *vio* operon. This plasmid was designated as pEC-J-vio2 (Fig. 2). Results were shown in (Fig. 1) that both biomass and violacein titer of *C. glutamicum* ATCC 21850 (pEC-J-vio2) were higher than pEC-J-vio1 (15 vs 20 g DCW L^−1^; 629 vs 815 mg L^−1^, respectively), so the introduction of complete RBS sequences of *C. glutamicum* to five structural *vio* genes improved both violacein production and host growth status. Our results proved that the compressed *vio* operon actually defected its expression in heterologous host, which might be a common problem in genetic and metabolic engineering. Construction of the large plasmid (14049 bps) from six parts was not an easy task, however, the improved production titer suggested that de novo construction of a pathway operon with completed synthetic RBSs is a functional strategy for solve such problems.

To further enhance violacein production, *vio* genes from another violacein natural producer, *C. violaceum*, were also individually cloned and assembled to form a similar synthetic *vio* operon as pEC-C-vio1. With this strategy, violacein production of the strain 21850 (pEC-C-vio1) in batch culture was further improved to reach 1116 mg L^−1^ (Fig. 1).

Inspired by previous success, we aimed to further optimized the modified *vio* operon. To investigate whether the gene order affects violacein production in *C. glutamicum*, a novel vio operon was constructed with a different order. The gene order in native violacein producers were all in P_*vio*_-vioA-vioB-vioC-vioD-vioE sequence, which was different from the order of violacein biosynthetic pathway. Tomoko Nishizaki [22] reordered five *crt* genes of the natural carotenoid cluster in *Pantoea ananatis* into the order of its metabolic pathway, and enhanced production of zeaxanthin in *E. coli*. In violacein biosynthesis pathway, the sequence of the reaction catalyzed by the enzymes is ABEDC. In addition, *vio*B was reported to be rate-limiting step enzyme [1], and Vio*D* is not evolved in formation deoxyviolacein. So that *vioD* gene was put in the last position of the operson. Thus, *vio* genes was reordered in a sequence of BAECD (Fig. 2), and plasmid pEC-C-vio2 was constructed. However, results showed that both biomass and violacein titer of 21850 (pEC-C-vio2) strain were lower than pEC-C-vio1 (30 vs 25 g DCW L^−1^; 1102 vs 629 mg L^−1^, respectively). This result suggested that the gene order we designed was not optimal than original one. There might be more optimal gene sequences for heterologous expression of *vio*, however, the enormous work associated with construction of all possible combinations made it impossible to find them. We are planning to study and try to develop some new methods to tackle this problem.

### Excretion of violacein by the high production strain ATCC 21850 (pEC-C-vio1)

**Fig. 3:**
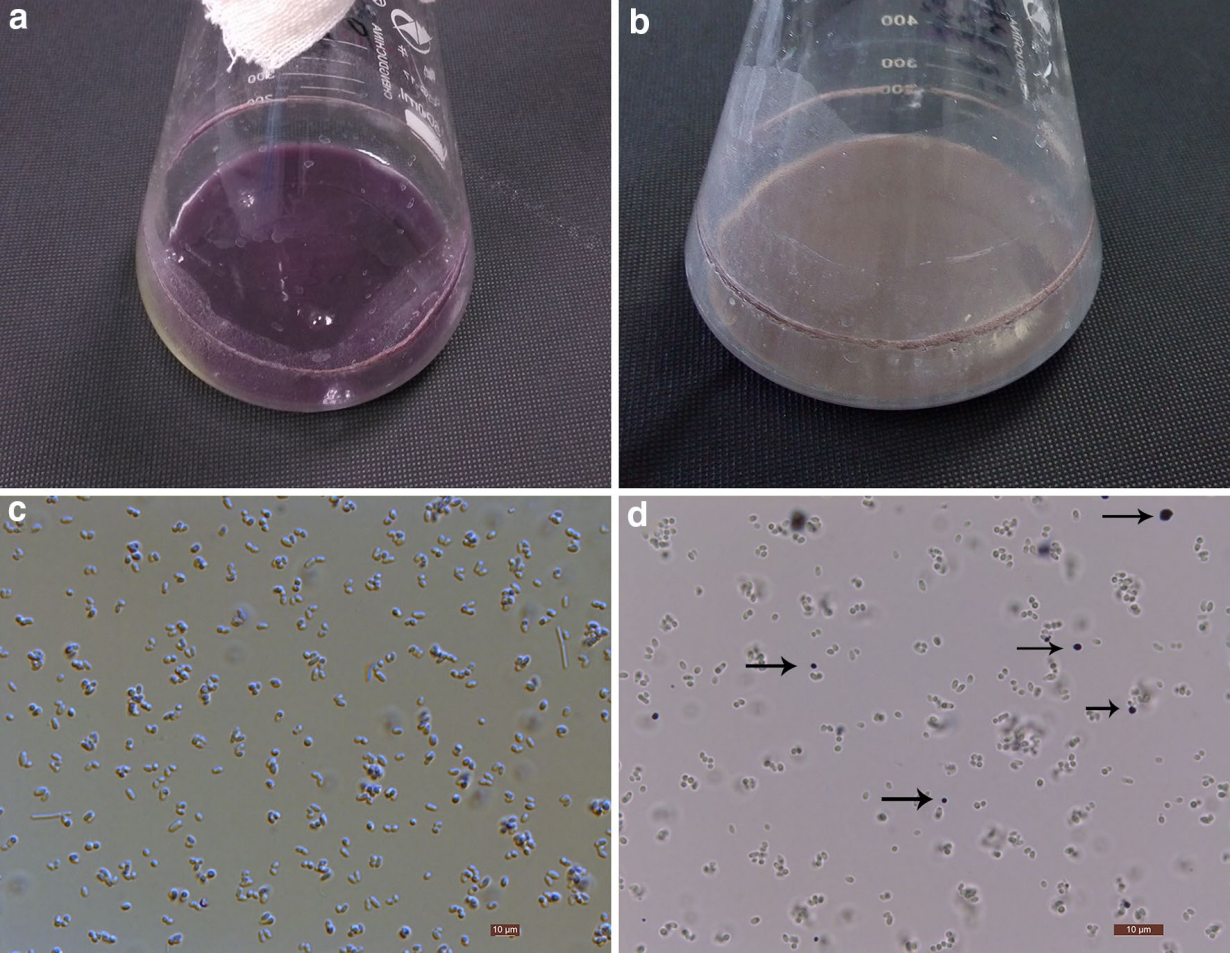
Violacein fermentation culture pictures and microscopic pictures. *C. glutamicum* 21850 (pEC-J-vio1) culture (**a**) and its corresponding microscopic photo (**c**); 21850 (pEC-C-vio1) culture (**b**) and its corresponding microscopic photos (**d**), extracellular crude violacein crystals are indicated with the *arrows*

In our study, the culture color of our highest production strain ATCC 21850 (pEC-C-vio1) turned into black. Meanwhile, the lower production strain, for example, ATCC 21850 (pEC-J-vio1) had a purple colored culture, as showed in Fig. 3. It was reported that high violacein producers, either native or heterologous, sometimes excreted violacein into culture [12]. To determine if our high production strain excreted crystalized violacein, microscope was used to observe the culture. The microscopic photos demonstrated that ATCC 21850 (pEC-C-vio1) produced extracellular purple particles similar to the images of previous report [12] which were most possibly violacein crystals, as indicated with arrows in Fig. 3d; while control strain ATCC 21850 (pEC-J-vio1) in the same condition produced no obvious extracellular particles. This microscopic phenomenon might explain the sharply different color between high and low production strains. The extracellular crystal phenotype also implies an economical downstream process for extraction and purification of produced violacein, giving advantages for industrializing this technology.

### Fermentation parameter optimization for violacein production

Fermentation is a complex process, many parameters needs to be studied and optimized to achieve good production. In our work, basic fermentation optimization was carried out to find optimal culture media, fermentation temperature, IPTG induction concentration and induction time.

**Fig. 4:**
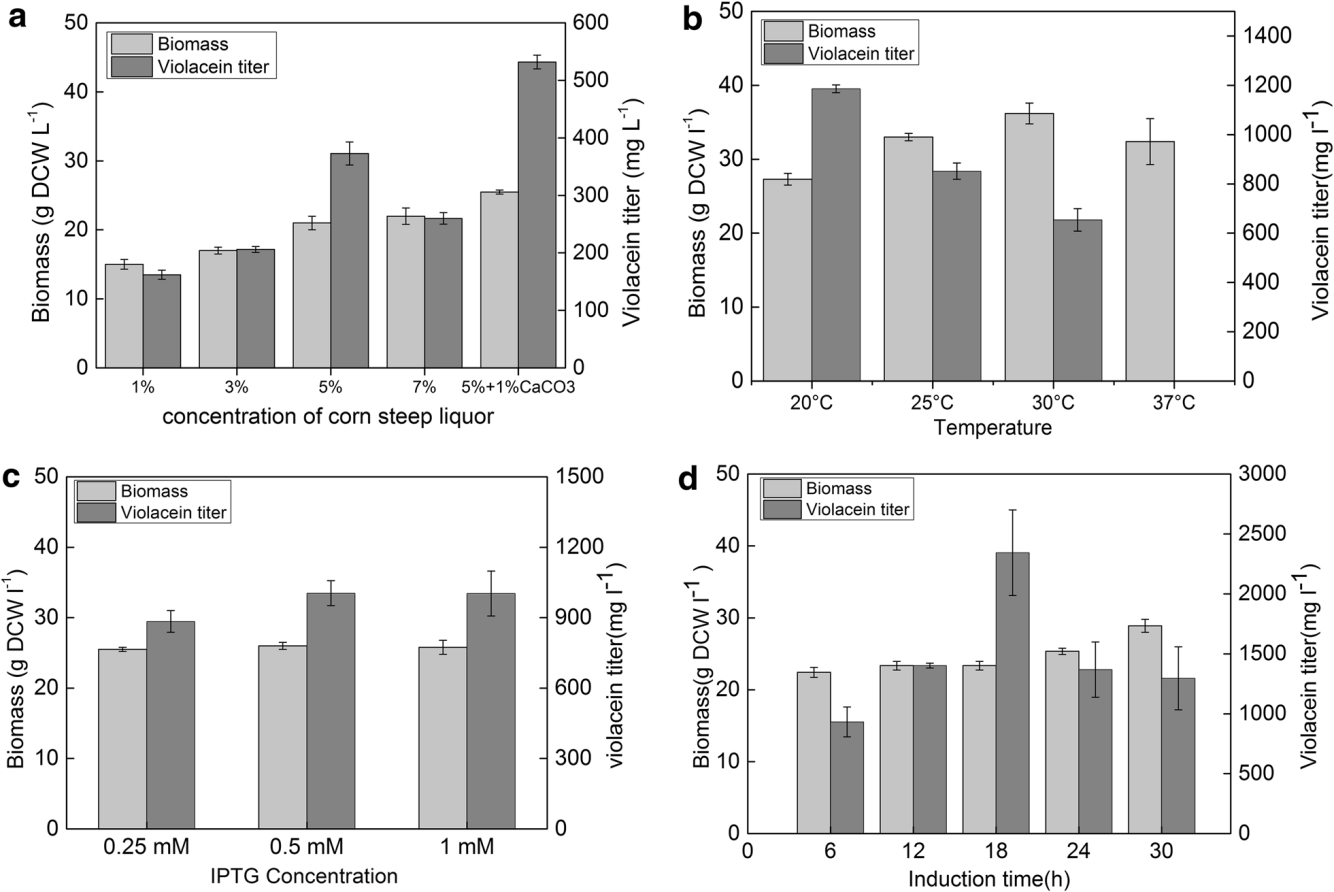
Fermentation parameter optimization. **a** Violacein production and biomass of 21850 (pEC-vioABCDE) with 1, 3, 5 and 7 % corn steep liquor supplementation in fermentation. 1 % CaCO_3_ was added to 5 % steep liquor culture for further improvement. **b** Temperature optimization for 21850 (pEC-J-vio1) of the second-phase of fermentation. violacein and biomass at 20, 25, 30, 37 °C was illustrated. **c** IPTG concentration was optimized for 21850 (pEC-J-vio1) fermentation at 0.25, 0.5, 1.0 mM for 21850 (pEC-J-vio1). Corresponding violacein production and biomass was illustrated. **d** Optimization of induction time of 21850 (pEC-C-vio1) for violacein production. After inoculation, 0.5 mM IPTG was added at 6, 12, 18, 24, 30 h and then fermentation was performed at 20 °C for 72 h with addition of IPTG. Experiments were carried out in triplicate

Fermentation medium is a key factor of *C. glutamicum* fermentation, in which corn steep liquor is a complex low-cost nitrogen source reported to significantly affect amino acid production [23]. Various concentrations were evaluated in term of violacein production to determine the optimum. To eliminate variations caused by induction concentration, induction time, induction growth status and so on, strain 21850 (pEC-vioABCDE) with constitutive expression of the *vio* operon was selected as testing strain. As illustrated in Fig. 4a, the highest violacein titer was obtained at 5 % corn steep liquor with addition of 1 % CaCO_3_.

Violacein production was reported to benefit from low temperature culturing, and typically the quantity of cell mass is also a major factor for fermentation production. To accumulate large amount of biomass before shifting to low temperature for violacein production, the classic two-phase fermentation process was employed. In this process, cells were cultured at 30 °C and then shifted to another temperature for fermentation product accumulation as previously reported [3, 10, 12, 24]. Here we performed experiments to determine an optimal temperature of the second phase fermentation for 21850 (pEC-C-vio1), selected from 37, 30, 25 and 20 °C (Fig. 4b). A trend of decreasing violacein production with higher temperature was observed, probably due to folding problems of heterologous enzymes at higher temperature [10]. Since the highest biomass was achieved at 30 °C, considering balance between cell growth and violacein production, 20 °C but not lower temperature was selected as the second phase temperature of fermentation.

Due to inducible promoter was employed for *vio* gene expression, concentrations of the inducer IPTG were studied for optimal fermentation condition of 21850 (pEC-C-vio1). whereas IPTG is toxic to host cell and increases fermentation cost when used at high concentration, lower concentrations might cause inadequate transcription induction [25]. In our work, various concentrations were applied and the optimum was determined to be 0.5 mM as shown in Fig. 4c. IPTG supplementation time is also a key parameter for optimized fermentation process. Theoretically, target enzymes are mainly produced after induction, so appropriate induction time enables balance between biomass accumulation and *vio* enzymes expression. As shown in Fig. 4d, different induction time was tested for fermentation, and it was determined that induction after 18 h yielded the highest violacein titer at 2344 mg L^−1^. This was also the highest titer reported in shake flask fermentation without tryptophan addition.

### Fed-batch fermentation of *C. glutamicum* ATCC 21850 (pEC-C-vio1) for violacein production

**Fig. 5:**
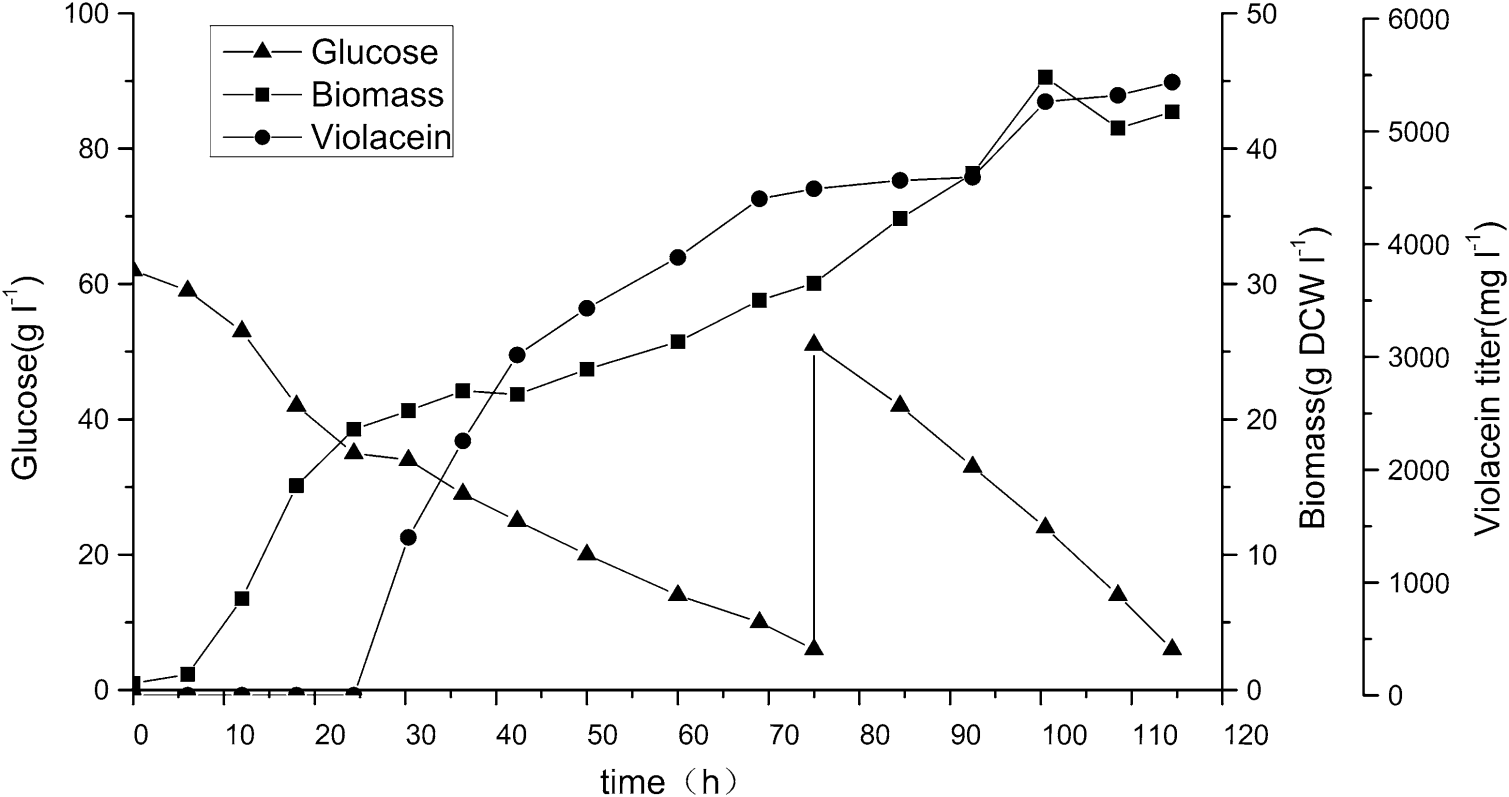
Fed-batch fermentation of violacein with *C. glutamicum* ATCC 21850 (pEC-C-vio1) in 3 L NBS BioFlo 115 fermenter

Bioreactor fermentation was carried out with the best production strain *C. glutamicum* ATCC 21850 (pEC-C-vio1) in a 3 L fermenter with the determined optimal parameters. As illustrated in Fig. 5, at 18 h after inoculation before induction, glucose consumption rate was about 1 g L^−1^ h^−1^, growth rate was 0.822 g DCW L^−1^ h^−1^ with no obvious lag phase. There was no violacein accumulation at this point. After induction, between 18 and 75 h, glucose consumption rate was about 0.6 g L^−1^ h^−1^ and growth rate decreased to 0.264 g DCW L^−1^ h^−1^ probably owing to change of temperature from 30 to 20 °C. within less than 6 h after induction, fermentation culture gradually became purple indicating initiation of violacein production. And violacein productivity from 24 to 75 h was measured to be 88 mg L^−1^ h^−1^. When glucose decreased to 6 g L^−1^, 51 g L^−1^ was fed to the reactor, which was consumed subsequently at a consumption rate of 1.1 g L^−1^ h^−1^. However, violacein productivity decreased at this stage probably due to product inhibition. Biomass reached 46.5 g DCW L^−1^ at 100 h, and began to decrease probably due to similar reason.

During the fermentation process, 101 g glucose was consumed and 5436 mg L^−1^ crude violacein was obtained with an overall productivity of 47 mg L^−1^ h^−1^. The highest titer reported was 1.75 g L^−1^ with a productivity of 36 mg L^−1^ h^−1^, which was achieved in Xing’s lab in 2015 [12]. Thus, Both production titer and productivity in this work are currently the highest from both native or heterologous hosts, but the yield at 0.054 g-vioalcein/g-glucose is lower than 0.116 reported previously [12].

## Conclusion

**Fig. 6:**
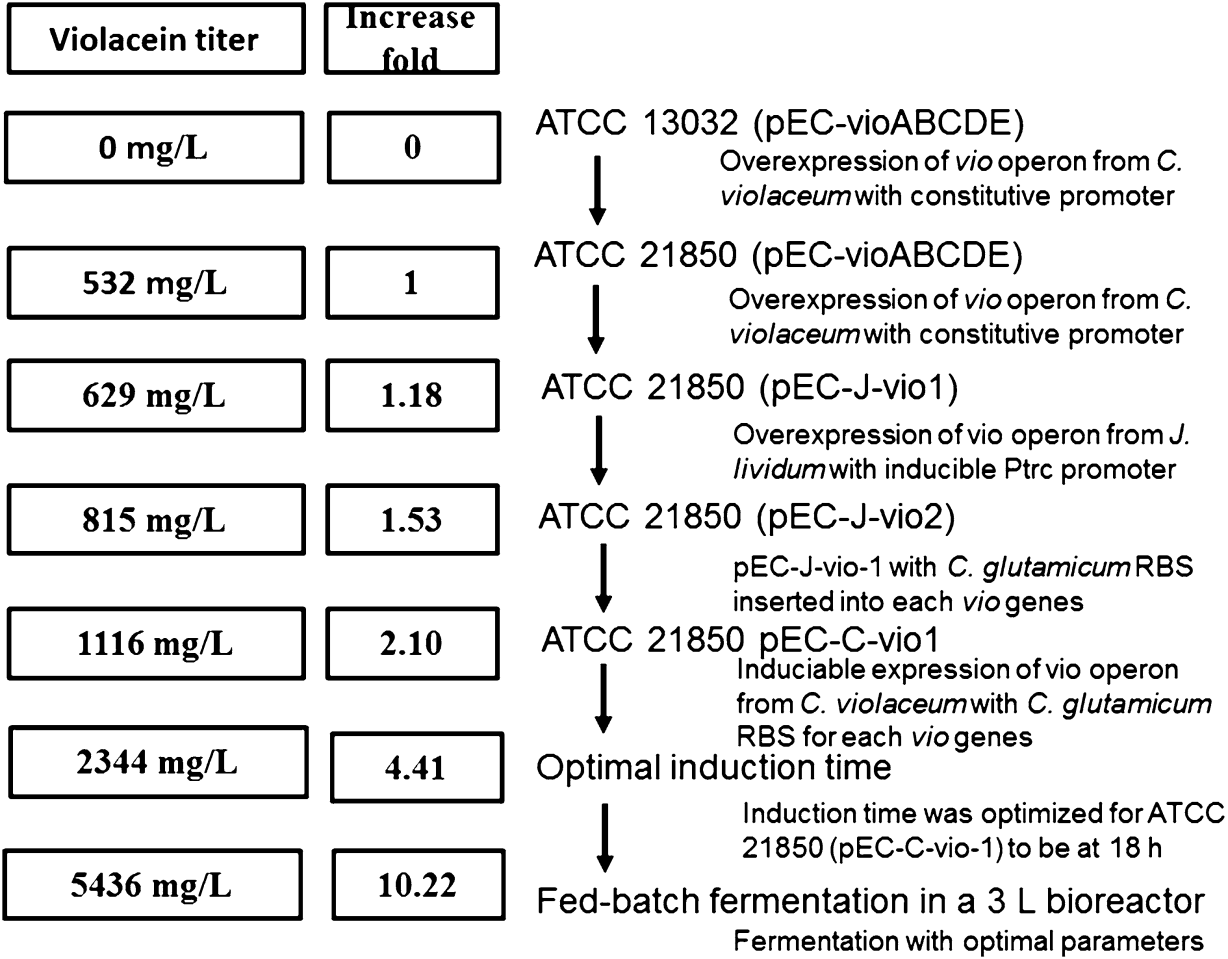
Summarization of metabolic engineering and fermentation optimization practice for successful violacein microbial cell factory construction

*Corynebacterium* was used as a metabolic engineering chassis for production of violacein, due to its GRAS status and advantages in tryptophan fermentation. With extensive metabolic engineering and fermentation optimization practice, *C. glutamicum* 21850 (pEC-C-vio1) was able to produce violacein with both titer and productivity at the highest level ever reported. In the engineering process, multiple engineering strategy was applied to the vio operon. To be specific, different promoters were applied; compressed genes in *vio* operon were artificially extended and rearranged in various sequences; and *vio* operons from different violacein producers were expressed and compared. In fermentation optimization part, four important parameters were studied, including culture media, temperature of two phase fermentation, induction concentration and induction time. With these effort, crude violacein production of *C. glutamicum* was increased from zero to a titer of 5436 mg L^−1^ as illustrated in Fig. 6. The titer and productivity we achieved in this work represent highest level to date. Future work may be focused on systematically metabolic engineering of the complete synthetic pathway and related metabolic network to further improve production, productivity and yield.

## Additional files

10.1186/s12934-016-0545-0 Strains, plasmids and oligonucleotides used in this study. **Figure S1.** The linear relationship between Absorbance at 570 nm a nd concentration of crude violacein. **Figure S2.** Batch cultivations of *C. glutamicum* in LBHIS broth.
10.1186/s12934-016-0545-0 Plasmid profiles and gene sequences.

## Abbreviations

GRAS: generally recognized as safe
LB: lysogeny broth
Trp: tryptophan
IPTG: isopropyl-β-d-thiogalactopyranoside
CSL: corn steep liquor
