# Supplementary material for: Engineering Corynebacterium glutamicum for violacein hyper production
Source: Microb Cell Fact. 2016 Aug 24;15(1):148. doi: 10.1186/s12934-016-0545-0 (PMC4997675; doi:10.1186/s12934-016-0545-0)
Supplement: Supplementary file 1 — 10.1186/s12934-016-0545-0 Strains, plasmids and oligonucleotides used in this study. Figure S1. The linear relationship between Absorbance at 570 nm a nd concentration of crude violacein. Figure S2. Batch cultivations of C. glutamicum in LBHIS broth. [file 12934_2016_545_MOESM1_ESM.docx]

**Supplementary information**

**The new host of *Corynebacterium glutamicum* for production of the crude violacein**

Hongnian Sun^1^, Dongdong Zhao^2^, Bin Xiong^2^, Chungzhi Zhang^1*^, Changhao Bi^2*^

^1^College of Biotechnology, Dalian Polytechnic University, Ministry of Education, Dalian 116034, P R China.

^2^Tianjin Institute of Industrial Biotechnology, Chinese Academy of Sciences, Tianjin 300308, P R China.

**Supplemental Table S1**. **Strains, plasmids, and oligonucleotides used in this study.**

| Strains/plasmids/primers | Relevant characteristics/sequences | Source/reference/notes |
| --- | --- | --- |
| **Strains** |  |  |
| *E. coli* DH5α | F^-^*endA1thi-1 recA1 relA1 gyrA96deoRΦ*80*dlac*Δ(*lac*Z) M15 Δ(*lacZYA-argF*)*U169hsdR17*(r_K_^-^, m_K_^+^) λ^–^*supE44 phoA* | Invitrogen |
| ATCC 13032 | *C. glutamicum* wild-type | ATCC |
| ATCC 21850 | 4-MT^r^ 5-MT^r^ 6-FT^r^ 4-Ap^r^ 4-FP^r^ TyrHx^r^ Phe^-^ Tyr^-^ | ATCC |
| **Plasmids** |  |  |
| pEC-XK99E | *C. glutamicum/E. coli* shuttle expression vector, Ptrc,lacIq,Kanr | Add gene |
| pEC-vioABCDE | derived from pEC-XK99E, constitutive expression of *C. violaceum* vio operon | This study |
| pEC-J-vio-1 | pEC-XK99E derivative containing vio operon from *J. lividum*, expressed under control of inducible promoter Ptrc | This study |
| pEC-J-vio-2 | pEC-XK99E derivative containing synthetic *J. lividum* vio operon with each gene containing complete *C. glutamicum* RBS sequence | This study |
| pEC-C-vio-1 | pEC-XK99E derivative containing synthetic *C. violaceum* vio operon with each gene containing complete *C. glutamicum* RBS sequence | This study |
| pEC-C-vio-2 | pEC-XK99E derivative containing synthetic *C. violaceum* vio operon with each gene containing complete *C. glutamicum* RBS sequence, which gene order changed to *vioB, vioA, vioE, vioC, vioD* | This study |
| pKMV-vioA | cloning vector in *E. coli*，Kanr，colE1 ori, containing *vioA* from *C. violaceum* | This study |
| pKMV-vioB | cloning vector in *E. coli*，Kanr，colE1 ori, containing *vioB* from *C. violaceum* | This study |
| pKMV-vioC | cloning vector in *E. coli*，Kanr，colE1 ori, containing *vioC* from *C. violaceum* | This study |
| pKMV-vioD | cloning vector in *E. coli*，Kanr，colE1 ori, containing *vioD* from *C. violaceum* | This study |
| pKMV-vioE | cloning vector in *E. coli*，Kanr，colE1 ori, containing *vioE* from *C. violaceum* | This study |
| **Oligonucleotides** |  |  |
| SD sequences | GAAAGGAGGTTTGGACA | This study |
| pEC-P1-F | CCAGGTCTCAAGCCGGATAAAACGAAAGGCTCAGTCG | This study |
| pEC-P1-R | CCAGGTCTCAGCATTCACCACCCTGAATTGACTCTCTTCCGG | This study |
| pEC-P2-F | CCAGGTCTCAATGCGCAACGCAATTAATGTGAGTTAGC | This study |
| pEC-P2-R | CCAGGTCTCATACGAAATTCCTTTTTAACGTTCACTGTTTCC | This study |
| C-vioA-F | CCAGGTCTCACGTATGAAGCACTCTTCTGACATCTGTATCG | This study |
| C-vioA-R | CCAGGTCTCAACCTCCTTTCCTATCTTCTCAAAGCAGCGATTCTTTGC | This study |
| C-vioB-F | CCAGGTCTCAAGGTTTGGACAATGAGCATTCTGGATTTCCCGCGT | This study |
| C-vioB-R | CCAGGTCTCACTCCTTTCCTATTAGGCCTCGCGGCTCAGT | This study |
| C-vioC-F | CCAGGTCTCAGGAGGTTTGGACAATGAAAAGAGCTATCATCGTTGGTGGTGG | This study |
| C-vioC-R | CCAGGTCTCAAAACCTCCTTTCCTAGTTAACTCTACCGATCTTGTACCAAACG | This study |
| C-vioD-F | CCAGGTCTCAGTTTGGACAATGAAAATCTTGGTTATCGGTGCTGGTCC | This study |
| C-vioD-R | CCAGGTCTCACCAAACCTCCTTTCCTATCTTTGCAAAGCGTATCTCAAGTTTTGAGCC | This study |
| C-vioE-F | CCAGGTCTCATTGGACAATGGAAAACAGAGAACCACCATTGTTGCC | This study |
| C-vioE-R | CCAGGTCTCAGGCTATCTCTTAGCAGCGAAAACAGCG | This study |
| J -pec1-F | CCAGGTCTCAGGATAAAACGAAAGGCTCAGTCGAAAG | This study |
| J -pec1-R | CCAGGTCTCACCTCGCTAGCGCGTTGCTGCTTCGC | This study |
| J -pec2-F | CCAGGTCTCAGAGGAAGAGCCAGAGCAGAAGG | This study |
| J -pec2-R | CCAGGTCTCACACTGTTTCCTGTGTGAAATTGTTATCCGC | This study |
| J -vio-F | TGAACGTTAAAAAGGAATTTCGTATGAAGCATTCTTCCGATATCTGCATTGTCGGC | This study |
| J- vio-R | TCGTTTTATCCGGCTAGCGCTTGGCGGCGAAGACG | This study |
| J-vioA-F | CCAGGTCTCAGTATGAGCACGTATTCTGACATTTGCATCG | This study |
| J-vioA-R | CCAGGTCTCAACCTCCTTTCTCATGCGCGCTCGGTCGAGG | This study |
| J-vioB-F | CCAGGTCTCAAGGTTTGGACAATGAGCCTACTTGACTTCCCCCGCC | This study |
| J-vioB-R | CCAGGTCTCACTCCTTTCTCAAGCCTCTCTTGACATCTTTCCCCG | This study |
| J-vioC-F | CCAGGTCTCAGGAGGTTTGGACAATGCATAAAATCATTATCGTCGGCGG | This study |
| J-vioC-R | CCAGGTCTCACAAACCTCCTTTCTTAATTTACCCTTCCAAGTTTGTACCA | This study |
| J-vioD-F | CCAGGTCTCATTTGGACAATGAAAATTCTCGTCATCGGCGCAGG | This study |
| J-vioD-R | CCAGGTCTCATTCTTAGCGGCCCAGCGCGTAG | This study |
| J-vioE-F | CCAGGTCTCAAGAAAGGAGGTTTGGACAATGCCGACACACGTCTCCCC | This study |
| J-vioE-R | CCAGGTCTCAGTCAGGTGTTGCAAGACGTAAAGAC | This study |
| C-pec-F | CCAGGTCTCAAGCCGGATAAAACGAAAGGCTCAGTCG | This study |
| C-pec-R | CCAGGTCTCAATACGAAATTCCTTTTTAACGTTCACTGTTTCC | This study |
| C-vio-F | CCAGGTCTCAGTATGAAGCACTCTTCTGACATCTGTATCG | This study |
| C-vio-R | CCAGGTCTCAGGCTATCTCTTAGCAGCGAAAACAGCG | This study |
| 2C-pec-F | CCAGGTCTCAAGCCGGATAAAACGAAAGGCTCAGTCG | This study |
| 2C-pec-R | CCAGGTCTCAATACGAAATTCCTTTTTAACGTTCACTGTTTCC | This study |
| 2C-vioB-F | CCAGGTCTCAAGCCGGATAAAACGAAAGGCTCAGTCG | This study |
| 2C-vioB-R | CCAGGTCTCAATACGAAATTCCTTTTTAACGTTCACTGTTTCC | This study |
| 2C-vioA-F | CCAGGTCTCAAGGTTTGGACAATGAAGCACTCTTCTGACATCTGTATCG | This study |
| 2C-vioA-R | CCAGGTCTCACTCCTTTCCTATCTTCTCAAAGCAGCGATTCTTTGC | This study |
| 2C-vioE-F | CCAGGTCTCAGGAGGTTTGGACAATGGAAAACAGAGAACCACCATTGTTGCC | This study |
| 2C-vioE-R | CCAGGTCTCACAAACCTCCTTTCCTATCTCTTAGCAGCGAAAACAGCG | This study |
| 2C-vioC-F | CCAGGTCTCATTTGGACAATGAAAAGAGCTATCATCGTTGGTGGTGG | This study |
| 2C-vioC-R | CCAGGTCTCATGTCCAAACCTCCTTTCCTAGTTAACTCTACCGATCTTGTACCAAACG | This study |
| 2C-vioD-F | CCAGGTCTCAGACAATGAAAATCTTGGTTATCGGTGCTGGTCC | This study |
| 2C-vioD-R | CCAGGTCTCAGGCTATCTTTGCAAAGCGTATCTCAAGTTTTGAGCC | This study |

**Supplemental Figure S1**

1. The correlation of Absorbance at 570nm and concentration of crude violacein.
2. The correlation of Absorbance at 600nm and concentration of

L-tryptophan





1. The correlation of Absorbance at 600nm and cell dry weight.


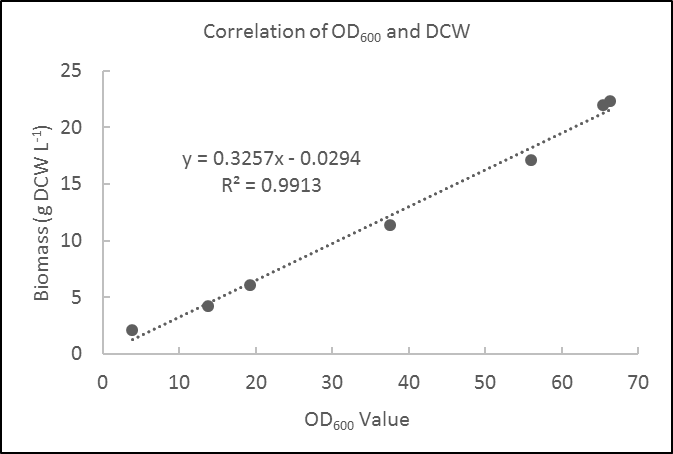


**Figure S2. Batch cultivations of C. glutamicum in LBHIS broth.**

Following activated cultivation in LBHIS at 30°C and 200 rpm for 24h, 4% of inoculum was transferred into 25 mL LBHIS broth and then was incubated for 4h at 30°C and 200 rpm followed by cultivating with 0.5 mM IPTG at 20°C for 48h.

**

**
