# Supplementary material for: Engineering Corynebacterium glutamicum for violacein hyper production
Source: Microb Cell Fact. 2016 Aug 24;15(1):148. doi: 10.1186/s12934-016-0545-0 (PMC4997675; doi:10.1186/s12934-016-0545-0)
Supplement: Supplementary file 2 — 10.1186/s12934-016-0545-0 Plasmid profiles and gene sequences. [file 12934_2016_545_MOESM2_ESM.docx]

**Supplementary plasmid profiles and gene sequences**

pEC-vioABCDE


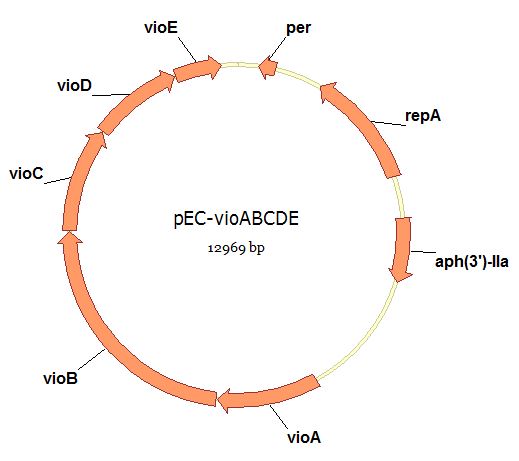


>pEC-vioABCDE

ctgttgtttgtcggtgaacgctctcctgagtaggacaaatccgccgggagcggatttgaacgttgcgaagcaacggcccggagggtggcgggcaggacgcccgccataaactgccaggcatcaaattaagcagaaggccatcctgacggatggcctttttgcgtttctacaaactctttttgtttatttttctaaatacattcaaatatgtatccgctcatgaattaattccgctagatgacgtgcggcttcgacctcctgggcgtggcgcttgttggcgcgctcgcggctggctgcggcacgacacgcgtctgagcagtattttgcgcgccgtcctcgtgggtcaggccggggtgggatcaggccaccgcagtaggcgcagctgatgcgatcctccactactgcgcgtcctcctggcgctgccgagcacgcagctcgtcggccagctcttcaaggtcggccacaagcgtttctaggtcgctcgcggcacttgcccagtcgcgtgatgctggcgcgtctgtcgtatcgagggcgcggaaaaatccgatcaccgtttttaaatcgacggcggcatcgagtgcgtcggactccagcgcgacatcggagagatccaccgctgatgcttcaggccagttttggtacttcgtcgtgaaggtcatgacaccattataacgaacgttcgttaaaaattctagccccaattctgataatttcttccggcactcctgcgaaaacctgcgagacttcttgcccagaaaaaacgccaagcgcagcggttaccgcactttttttccaggtgatttcaccctgaccagcgaagcggcactttagtgcatgaggtgtgcccctggtttcccctctttggagggttcaacccaaaaaagcacacaagcaaaaatgaaaatcatcatgagcaagttggtgcgaagcagcaacgcgctagcgaggaagagccagagcagaaggcggggaaccgttctctgccgacagcgtgagccccccttaaaaatcaggccggggaggaaccggggagggatcagagctaggagcgagacaccctaaagggggggaaccgttttctgctgacggtgtttcgtttattagttttcagcccgtggatagcggagggtgagggcaagtgagagccagagcaaggacgggacccctaaaggggggaaccgttttctgctgacggtgtttcgtttattagttttcagcccgtggacggccgcgtttagcttccattccaagtgcctttctgacttgttggatgcgcctttcactgacacctagttcgcctgcaagctcacgagtcgagggatcagcaaccgattgagaacgggcatccaggatcgcagttttgacgcgaagttcgagcaactcgcctgtcatttctcggcgtttgtttgcttccgctaatcgctgtcgcgtctcctgcgcatacttactttctgggtcagcccatctgcgtgcattcgatgtagctgcgccccgtcgccccatcgtcgctagagctttccgccctcggctgctctgcgtttccacccgacgagcagggacgactggctggcctttagccacgtagccgcgcacacgacgcgccatcgtcaggcgatcacgcatggcgggaagatccggctcccggccgtctgcaccgaccgcctgggcaacgttgtacgccacttcatacgcgtcgatgatcttggcatcttttaggcgctcaccagcagctttgagctggtatcccacggtcaacgcgtggcgaaacgcggtctcgtcgcgcgctcgctctggatttgtccagagcactcgcacgccgtcgatcaggtcgccggacgcgtccagggcgctcggcaggctcgcgtccaaaatcgctagcgccttggcttctgcggtggcgcgttgtgccgcttcaatgcgggcgcgtccgctggaaaagtcctgctcaatgtactttttcggcttctgtgatccggtcatcgttcgagcaatctccattaggtcggccagccgatccacacgatcatgctggcagtgccatttataggctgtcggatcgtctgagacgtgcagcggccaccggctcagcctatgcgaaaaagcctggtcagcgccgaaaacacgagtcatttcttccgtcgttgcagccagcaggcgcatatttgggctggttttacctgctgcggcatacaccgggtcaatgagccagatgagctggcatttcccgctcagcggattcacgccgatccaagccggcgctttttctaggcgtgcccatttctctaaaatcgcgtagacctgcgggtttacgtgctcaatcttcccgccggcctggtggctgggcacatcgatgtcaagcacgatcaccgcggcatgttgcgcgtgcgtcagcgcaacgtactggcaccgcgtcagcgcttttgagccagcccggtagagctttggttgggtttcgccggtatccgggtttttaatccaggcgctcgcgaaatctcttgtcttgctgccctggaagctttcgcgtcccaggtgagcgagcagttcgcggcgatcttctgccgtccagccgcgtgagccgcagcgcatagcttcggggtgggtgtcgaacagatcggcggacaatttccacgcgctagctgtgactgtgtcctgcggatcggctagagtcatgtcttgagtgctttctcccagctgatgactgggggttagccgacgccctgtgagttcccgctcacggggcgttcaactttttcaggtatttgtgcagcttatcgtgttttcttcgtaaatgaacgcttaactaccttgttaaacgtggcaaataggcaggattgatggggatctagcttcacgctgccgcaagcactcagggcgcaagggctgctaaaggaagcggaacacgtagaaagccagtccgcagaaacggtgctgaccccggatgaatgtcagctactgggctatctggacaagggaaaacgcaagcgcaaagagaaagcaggtagcttgcagtgggcttacatggcgatagctagactgggcggttttatggacagcaagcgaaccggaattgccagctggggcgccctctggtaaggttgggaagccctgcaaagtaaactggatggctttcttgccgccaaggatctgatggcgcaggggatcaagatctgatcaagagacaggatgaggatcgtttcgcatgattgaacaagatggattgcacgcaggttctccggccgcttgggtggagaggctattcggctatgactgggcacaacagacaatcggctgctctgatgccgccgtgttccggctgtcagcgcaggggcgcccggttctttttgtcaagaccgacctgtccggtgccctgaatgaactccaagacgaggcagcgcggctatcgtggctggccacgacgggcgttccttgcgcagctgtgctcgacgttgtcactgaagcgggaagggactggctgctattgggcgaagtgccggggcaggatctcctgtcatctcaccttgctcctgccgagaaagtatccatcatggctgatgcaatgcggcggctgcatacgcttgatccggctacctgcccattcgaccaccaagcgaaacatcgcatcgagcgagcacgtactcggatggaagccggtcttgtcgatcaggatgatctggacgaagagcatcaggggctcgcgccagccgaactgttcgccaggctcaaggcgcggatgcccgacggcgaggatctcgtcgtgacccatggcgatgcctgcttgccgaatatcatggtggaaaatggccgcttttctggattcatcgactgtggccggctgggtgtggcggaccgctatcaggacatagcgttggctacccgtgatattgctgaagagcttggcggcgaatgggctgaccgcttcctcgtgctttacggtatcgccgctcccgattcgcagcgcatcgccttctatcgccttcttgacgagttcttctgagcgggactctggggttcgcggaatcatgaccaaaatcccttaacgtgagttttcgttccactgagcgtcagaccccgtagaaaagatcaaaggatcttcttgagatcctttttttctgcgcgtaatctgctgcttgcaaacaaaaaaaccaccgctaccagcggtggtttgtttgccggatcaagagctaccaactctttttccgaaggtaactggcttcagcagagcgcagataccaaatactgtccttctagtgtagccgtagttaggccaccacttcaagaactctgtagcaccgcctacatacctcgctctgctaatcctgttaccagtggctgctgccagtggcgataagtcgtgtcttaccgggttggactcaagacgatagttaccggataaggcgcagcggtcgggctgaacggggggttcgtgcacacagcccagcttggagcgaacgacctacaccgaactgagatacctacagcgtgagctatgagaaagcgccacgcttcccgaagggagaaaggcggacaggtatccggtaagcggcagggtcggaacaggagagcgcacgagggagcttccagggggaaacgcctggtatctttatagtcctgtcgggtttcgccacctctgacttgagcgtcgatttttgtgatgctcgtcaggggggcggagcctatggaaaaacgccagcaacgcggcctttttacggttcctggccttttgctggccttttgctcacatgttctttcctgcgttatcccctgattctgtggataaccgtattaccgcctttgagtgagctgataccgctcgccgcagccgaacgaccgagcgcagcgagtcagtgagcgaggaagcggaagagcgcctgatgcggtattttctccttacgcatctgtgcggtatttcacaccgcatatggtgcactctcagtacaatctgctctgatgccgcatagttaagccagtatacactccgctatcgctacgtgactgggtcatggctgcgccccgacacccgccaacacccgctgacgcgccctgacgggcttgtctgctcccggcatccgcttacagacaagctgtgaccgtctccgggagctgcatgtgtcagaggttttcaccgtcatcaccgaaacgcgcgaggcagcagatcaattcgcgcgcgaaggcgaagcggcatgcatttacgttgacaccatcgaatggtgcaaaacctttcgcggtatggcatgatagcgcccggaagagagtcaattcagggtggtgaagcgcaacgcaattaatgtgagttagcgcgaattgatctggtttgacagcttatcatcgactgcacggtgcaccaatgcttctggcgtcaggcagccatcggaagctgtggtatggctgtgcaggtcgtaaatcactgcataattcgtgtcgctcaaggcgcactcccgttctggataatgttttttgcgccgacatcataacggttctggcaaatattctgaaatgagctgttgacaattaatcatccggctcgtataatgtgtggaattgtgagcggataacaatttcacacaggaaacagtgaacgttaaaaaggaatttcgtatgaagcattcttccgatatctgcattgtcggcgccggcatcagcggcctgacctgcgccagccatctgctcgactcgcccgcttgccgcggcctgtcgctgcgcatcttcgacatgcagcaggaggcgggcggccgcatccgctcgaagatgctggatggcaaggcgtcgatagagctgggcgcggggcgatactccccgcagctgcacccgcatttccagagcgcgatgcagcattacagccagaagagcgaggtgtatccgttcacccagctgaaattcaagagccatgtccagcagaagctgaagcgggcgatgaacgagttgtcgcccaggctgaaagagcatggcaaggaatcctttctccagttcgtcagccgctaccagggccatgacagcgcggtgggcatgatccgctccatgggctacgacgcgctgttcctgcccgacatctcggccgagatggcctacgacatcgtcggcaagcacccggaaatccagagcgtgaccgataacgacgccaaccagtggttcgcggcggaaacgggctttgcgggcctgatccagggcatcaaggccaaggtcaaggctgccggcgcgcgcttcagcctgggttaccggctgctgtcggtgaggacggacggcgacggctacctgctgcaactggccggcgacgacggctggaagctggaacaccggacccgccatctgatcctggccattcctccgtcggcgatggccgggctcaatgtcgacttccccgaggcgtggagcggcgcgcgctacggctcgctgccgctgttcaagggtttcctcacctacggcgagccatggtggctggactacaagctggacgaccaggtgctgatcgtcgacaacccgctgcgcaagatctacttcaagggcgacaagtacctgttcttctacaccgacagcgagatggccaattactggcgcggctgcgtggccgaaggagaggacggctacctggagcagatccgcacccatctggccagcgcgctgggcatcgttcgcgagcgcattccccagcccctcgcccatgtgcacaagtattgggcgcatggcgtggagttctgccgcgacagcgatatcgaccatccgtccgcgctcagccaccgcgacagcggcatcatcgcctgttcggacgcctacaccgagcactgcggctggatggagggcggcctgctcagcgcccgcgaagccagccgtctgctgctgcagcgcatcgccgcgtgaacggtccggccgccgcatcgcgtcgccgcccggttccgggcggcgcttgtcagccatgaccgttcgggaaacacatgagcattctggattttccacgcatccatttccgcggctgggcgcgggtcaacgcgcccaccgccaaccgcgatccgcacggccacatcgacatggccagcaatacggtggccatggcaggcgaaccgttcgacctcgcgcgccatccgaccgagttccaccgccacctgcggtcgctggggccgcgtttcggcctggacggccgggctgacccggaagggccgttcagcctggccgagggctacaacgcggccggcaacaaccatttctcctgggagagcgccaccgtcagccacgtgcagtgggatggcggcgaagcggaccgcggcgacggcctggtcggcgccaggctggcgctgtgggggcattacaacgattacctgcgcaccaccttcaaccgcgcgcgctgggtggacagcgaccccacccgccgcgacgcggcgcagatctacgccgggcagttcacgatcagcccggccggcgccggaccgggcacgccctggctgttcaccgccgacatcgacgacagccacggcgcgcgctggacgcgcggcggccacatcgccgagcgcggcggccatttcctggacgaggagttcggcctggcgcggctgttccagttctcggtgcccaaagaccatccgcacttcctgttccacccggggccattcgattccgaagcctggcgcaggctgcagctggcgctggaggacgacgacgtgctcggcctgacggtgcagtacgcgctgttcaatatgtcgacgccgccgcaacccaactcgccggtgttccacgacatggtcggcgtggtcggcctgtggcggcgcggcgaactggccagctacccggccggccggctgctgcgtccgcgccagcccgggctgggcgatctgacgctgcgcgtaagcggcggccgcgtggcgctgaatctggcctgcgccattccgttctccacccgggcggcgcagccgtccgcgccggacaggctgacgcccgatctcggggccaagctgccgttgggcgacctgctgctgcgcgacgaggacggcgcgttgctggcgcgggtgccgcaggcgctttaccaggattactggacgaaccacggcatcgtcgacctgccgctgctgcgcgagcccaggggctcgctgacgctgtccagcgagctggccgaatggcgcgagcaggactgggtcacgcagtccgacgcctccaatctttatttggaagcgccggaccgccgccacggccgtttctttccggaaagcatcgcgctgcgcagctatttccgcggcgaggcccgcgcgcgcccggacattccccaccggatcgaggggatgggtctggtcggcgtggagtcgcgccaggacggcgatgccgccgaatggcggctgaccggcctgcggcccggcccggcgcgcatcgtgctcgacgacggcgcggaggcgatcccgctgcgggtgctgccggacgactgggcgttggacgacgcgacggtggaggaggtcgattacgccttcctgtaccggcacgtgatggcctattacgagctggtctacccgttcatgtccgacaaggtgttcagcctggccgaccgctgcaagtgcgagacctacgccaggctgatgtggcagatgtgcgatccgcagaaccggaacaagagctactacatgcccagcacccgcgagctgtcggcgcccaaggccaggctgttcctcaaatacctggcccatgtcgagggccaggccaggctgcaggcgccgccgccggccgggccggcgcgcatcgagagcaaggcccagctggcggccgagctgcgcaaggcggtggatctggagttgtcggtgatgctgcagtacctgtacgccgcctattccattcccaattacgcccagggccagcagcgggtgcgcgacggcgcgtggacggcggagcagctgcagctggcctgcggcagcggcgaccggcgccgcgacggcggcatccgcgccgcgctgctggagatcgcccacgaggagatgatccattacctggtggtcaacaacctgctgatggcgctgggcgagccgttctacgccggcgtgccgctgatgggcgaggcggcgcggcaggcgttcggcctggacaccgaattcgcgctggagccgttctccgagtcgacgctggcgcgcttcgtccggctggaatggccgcacttcatccctgcgccgggcaaatccatcgccgactgctacgccgccatccgccaggcctttctcgatctgcccgacctgttcggcggcgaggccggcaagcgcggcggcgagcaccacttgttcctcaacgagctgaccaaccgcgcccatcccggctaccagctggaggtgttcgatcgcgacagcgcgctgttcggcatcgccttcgtcaccgaccagggcgagggcggggcgctggactcgccgcattacgagcattcgcatttccagcggctgcgggagatgtcggccaggatcatggcgcagtccgcgccgttcgagccggcgttgccggcgctgcgcaacccggtgctggacgagtcgccgggctgccagcgcgtggcggacggacgggcgcgcgcgctgatggcgctgtaccagggcgtgtacgagctgatgttcgcgatgatggcgcagcacttcgcggtcaagccgctgggcagcctcaggcgctcgcggctgatgaacgcggcgatcgacctgatgaccggcctgctcaggccgctgtcctgcgcgctgatgaacctgccgtcgggcatcgccggacgcaccgccgggccgccgctgccggggccggtggatacccgcagctacgacgactacgcgctgggctgccggatgctggcgcggcgctgcgagcgcctgctggagcaggcgtcgatgctggagccgggctggctgcccgacgcgcaaatggaactgctggatttctaccgccggcagatgctggatttggcttgtggaaagctttctagagaggcctgaaatgaaaagagcaatcatagtcggaggcgggctcgccggcgggctgaccgccatctacctggcgaagcgcggctacgaggtccacgtggtggaaaagcgcggcgacccgctgcgggacctgtcttcctacgtggatgtggtcagctcgcgggcgataggcgtcagcatgaccgtgcgcggcatcaagtcggtgctggcggccggcattccgcgcgcggagctggacgcctgcggcgaacccatcgtggcgatggcgttttccgtcggcggccagtaccggatgcgggagctcaagccgctggaggatttccgcccgctgtcgctgaaccgcgcggcgtttcagaagctgctgaacaagtacgccaacctggccggcgtccgctactacttcgagcacaagtgcctggacgtggatctggacggcaagtcggtgctgatccagggcaaggacggccagccgcagcgcttgcagggcgatatgatcatcggcgccgacggcgcgcactcggcggtgcggcaggcgatgcagagcgggttgcgccgcttcgaattccagcagactttcttccgccacggctacaagacgctggtgctgccggacgcgcaggcgctgggctaccgcaaggacacgctgtatttcttcggcatggactccggcggcctgttcgccggccgcgccgccaccatcccggacggcagcgtcagcatcgcggtctgcctgccgtacagcggcagccccagcctgaccaccaccgacgagccgacgatgcgcgcctttttcgaccgttacttcggcggcctgccgcgggacgcgcgcgacgagatgctgcgccagttcctggccaagcccagcaacgacctgatcaacgtccgttccagcaccttccactacaagggcaatgtgctgctgctgggcgacgccgcccacgccaccgcgcctttcctcggccagggcatgaacatggcgctggaggacgcgcgcaccttcgtcgagctgctggaccgccaccagggcgaccaggacaaggcctttcccgagttcaccgagctgcgcaaggtgcaggccgacgcgatgcaggacatggcgcgcgccaactacgacgtgctcagctgctccaatcccatcttcttcatgcgggcccgctacacccgctacatgcatagcaagtttcccggcctttacccgccggacatggcggagaagctgtacttcacgtccgagccgtacgacagactgcagcagatccagagaaaacagaacgtttggtacaagatagggagggtcaactgatgaagattctggtcatcggcgcggggccggccggcctggtgttcgccagccaactgaaacaggcgcgtccgctgtgggcgatagacatcgtcgaaaagaacgacgagcaggaagtgctgggctggggcgtggtgctgcccggccggcccggccagcatccggccaatccgctgtcctacctggacgcgccggagaggctgaatccgcagttcctggaagacttcaagctggtccaccacaacgagcccagcctgatgagcaccggcgtgctgctgtgcggcgtggagcgccgcggcctggtgcacgccttgcgcgacaagtgccgctcgcagggcatcgccatccgcttcgaatcgccgctgctggagcatggcgagctgccgctggccgactacgacctggtggtgctggccaacggcgtcaatcacaagaccgcccacttcaccgaggcgctggtgccgcaggtggactacggccgcaacaagtacatctggtacggcaccagccagctgttcgaccagatgaacctggtgttccgcacccacggcaaggacattttcatcgcccacgcctacaagtactcggacacgatgagcaccttcatcgtcgagtgcagcgaggagacctatgcccgcgcccgcctgggcgagatgtcggaagaggcgtcggccgaatacgtcgccaaggtgttccaggccgagctgggcggccacggcctggtgagccagcccggcctcggctggcgcaacttcatgaccctgagccacgaccgctgccacgacggcaagctggtgctgctgggcgacgcgctgcagtccggccacttctccatcggccacggcaccacgatggcggtggtggtggcgcagctgctggtgaaggcgctgtgcaccgaggacggcgtgccggccgcgctgaagcgcttcgaggagcgcgcgctgccgctggtccagctgttccgcggccatgccgacaacagccgggtctggttcgagacggtggaggagcgcatgcacctgtccagcgccgagttcgtgcagagcttcgacgcgcgccgcaagtcgctgccgccgatgccggaagcgctggcgcagaacctgcgctacgcgctgcaacgctgaggaggccgcatggaaaaccgggaaccgccgctgctgccggcgcgctggagcagcgcctatgtgtcgtactggagtccgatgctgccggatgaccagctgacgtccggctactgctggttcgactacgagcgcgacatctgtcggatagacggcctgttcaatccctggtcggagcgcgacaccggctaccggctgtggatgtccgaggtcggcaacgccgccagcggccgcacctggaagcagaaggtggcctatggccgcgagcggaccgccctgggcgagcagctgtgcgagcggccgctggacgacgagaccggcccgttcgccgagctgttcctgccgcgcgacgtgctgcgccggctgggcgcccgccatatcggccgccgcgtggtgctgggcagggaagccgacggctggcgctaccagcgtccgggcaaggggccgtccacgttgtacctggacgccgccagcggtacgccgctgaggatggtgaccggggacgaggcgtcgcgcgcgtcgctgcgcgatttccccaacgtcagcgaggccgagattcccgacgccgtcttcgccgccaagcgctagccggataaaacgaaaggctcagtcgaaagactgggcctttcgttttat

pEC-J-vio-1


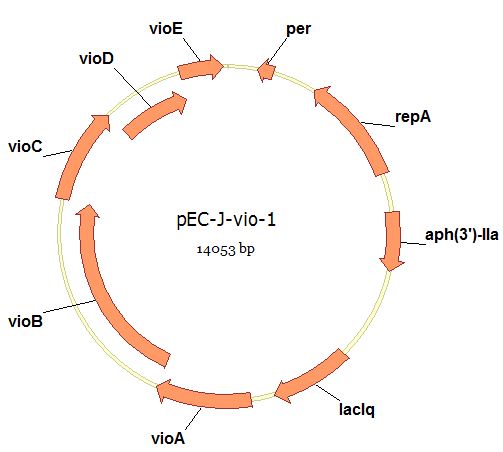


>pEC-J-vio-1

ctgttgtttgtcggtgaacgctctcctgagtaggacaaatccgccgggagcggatttgaacgttgcgaagcaacggcccggagggtggcgggcaggacgcccgccataaactgccaggcatcaaattaagcagaaggccatcctgacggatggcctttttgcgtttctacaaactctttttgtttatttttctaaatacattcaaatatgtatccgctcatgaattaattccgctagatgacgtgcggcttcgacctcctgggcgtggcgcttgttggcgcgctcgcggctggctgcggcacgacacgcgtctgagcagtattttgcgcgccgtcctcgtgggtcaggccggggtgggatcaggccaccgcagtaggcgcagctgatgcgatcctccactactgcgcgtcctcctggcgctgccgagcacgcagctcgtcggccagctcttcaaggtcggccacaagcgtttctaggtcgctcgcggcacttgcccagtcgcgtgatgctggcgcgtctgtcgtatcgagggcgcggaaaaatccgatcaccgtttttaaatcgacggcggcatcgagtgcgtcggactccagcgcgacatcggagagatccaccgctgatgcttcaggccagttttggtacttcgtcgtgaaggtcatgacaccattataacgaacgttcgttaaaaattctagccccaattctgataatttcttccggcactcctgcgaaaacctgcgagacttcttgcccagaaaaaacgccaagcgcagcggttaccgcactttttttccaggtgatttcaccctgaccagcgaagcggcactttagtgcatgaggtgtgcccctggtttcccctctttggagggttcaacccaaaaaagcacacaagcaaaaatgaaaatcatcatgagcaagttggtgcgaagcagcaacgcgctagcgaggaagagccagagcagaaggcggggaaccgttctctgccgacagcgtgagccccccttaaaaatcaggccggggaggaaccggggagggatcagagctaggagcgagacaccctaaagggggggaaccgttttctgctgacggtgtttcgtttattagttttcagcccgtggatagcggagggtgagggcaagtgagagccagagcaaggacgggacccctaaaggggggaaccgttttctgctgacggtgtttcgtttattagttttcagcccgtggacggccgcgtttagcttccattccaagtgcctttctgacttgttggatgcgcctttcactgacacctagttcgcctgcaagctcacgagtcgagggatcagcaaccgattgagaacgggcatccaggatcgcagttttgacgcgaagttcgagcaactcgcctgtcatttctcggcgtttgtttgcttccgctaatcgctgtcgcgtctcctgcgcatacttactttctgggtcagcccatctgcgtgcattcgatgtagctgcgccccgtcgccccatcgtcgctagagctttccgccctcggctgctctgcgtttccacccgacgagcagggacgactggctggcctttagccacgtagccgcgcacacgacgcgccatcgtcaggcgatcacgcatggcgggaagatccggctcccggccgtctgcaccgaccgcctgggcaacgttgtacgccacttcatacgcgtcgatgatcttggcatcttttaggcgctcaccagcagctttgagctggtatcccacggtcaacgcgtggcgaaacgcggtctcgtcgcgcgctcgctctggatttgtccagagcactcgcacgccgtcgatcaggtcgccggacgcgtccagggcgctcggcaggctcgcgtccaaaatcgctagcgccttggcttctgcggtggcgcgttgtgccgcttcaatgcgggcgcgtccgctggaaaagtcctgctcaatgtactttttcggcttctgtgatccggtcatcgttcgagcaatctccattaggtcggccagccgatccacacgatcatgctggcagtgccatttataggctgtcggatcgtctgagacgtgcagcggccaccggctcagcctatgcgaaaaagcctggtcagcgccgaaaacacgagtcatttcttccgtcgttgcagccagcaggcgcatatttgggctggttttacctgctgcggcatacaccgggtcaatgagccagatgagctggcatttcccgctcagcggattcacgccgatccaagccggcgctttttctaggcgtgcccatttctctaaaatcgcgtagacctgcgggtttacgtgctcaatcttcccgccggcctggtggctgggcacatcgatgtcaagcacgatcaccgcggcatgttgcgcgtgcgtcagcgcaacgtactggcaccgcgtcagcgcttttgagccagcccggtagagctttggttgggtttcgccggtatccgggtttttaatccaggcgctcgcgaaatctcttgtcttgctgccctggaagctttcgcgtcccaggtgagcgagcagttcgcggcgatcttctgccgtccagccgcgtgagccgcagcgcatagcttcggggtgggtgtcgaacagatcggcggacaatttccacgcgctagctgtgactgtgtcctgcggatcggctagagtcatgtcttgagtgctttctcccagctgatgactgggggttagccgacgccctgtgagttcccgctcacggggcgttcaactttttcaggtatttgtgcagcttatcgtgttttcttcgtaaatgaacgcttaactaccttgttaaacgtggcaaataggcaggattgatggggatctagcttcacgctgccgcaagcactcagggcgcaagggctgctaaaggaagcggaacacgtagaaagccagtccgcagaaacggtgctgaccccggatgaatgtcagctactgggctatctggacaagggaaaacgcaagcgcaaagagaaagcaggtagcttgcagtgggcttacatggcgatagctagactgggcggttttatggacagcaagcgaaccggaattgccagctggggcgccctctggtaaggttgggaagccctgcaaagtaaactggatggctttcttgccgccaaggatctgatggcgcaggggatcaagatctgatcaagagacaggatgaggatcgtttcgcatgattgaacaagatggattgcacgcaggttctccggccgcttgggtggagaggctattcggctatgactgggcacaacagacaatcggctgctctgatgccgccgtgttccggctgtcagcgcaggggcgcccggttctttttgtcaagaccgacctgtccggtgccctgaatgaactccaagacgaggcagcgcggctatcgtggctggccacgacgggcgttccttgcgcagctgtgctcgacgttgtcactgaagcgggaagggactggctgctattgggcgaagtgccggggcaggatctcctgtcatctcaccttgctcctgccgagaaagtatccatcatggctgatgcaatgcggcggctgcatacgcttgatccggctacctgcccattcgaccaccaagcgaaacatcgcatcgagcgagcacgtactcggatggaagccggtcttgtcgatcaggatgatctggacgaagagcatcaggggctcgcgccagccgaactgttcgccaggctcaaggcgcggatgcccgacggcgaggatctcgtcgtgacccatggcgatgcctgcttgccgaatatcatggtggaaaatggccgcttttctggattcatcgactgtggccggctgggtgtggcggaccgctatcaggacatagcgttggctacccgtgatattgctgaagagcttggcggcgaatgggctgaccgcttcctcgtgctttacggtatcgccgctcccgattcgcagcgcatcgccttctatcgccttcttgacgagttcttctgagcgggactctggggttcgcggaatcatgaccaaaatcccttaacgtgagttttcgttccactgagcgtcagaccccgtagaaaagatcaaaggatcttcttgagatcctttttttctgcgcgtaatctgctgcttgcaaacaaaaaaaccaccgctaccagcggtggtttgtttgccggatcaagagctaccaactctttttccgaaggtaactggcttcagcagagcgcagataccaaatactgtccttctagtgtagccgtagttaggccaccacttcaagaactctgtagcaccgcctacatacctcgctctgctaatcctgttaccagtggctgctgccagtggcgataagtcgtgtcttaccgggttggactcaagacgatagttaccggataaggcgcagcggtcgggctgaacggggggttcgtgcacacagcccagcttggagcgaacgacctacaccgaactgagatacctacagcgtgagctatgagaaagcgccacgcttcccgaagggagaaaggcggacaggtatccggtaagcggcagggtcggaacaggagagcgcacgagggagcttccagggggaaacgcctggtatctttatagtcctgtcgggtttcgccacctctgacttgagcgtcgatttttgtgatgctcgtcaggggggcggagcctatggaaaaacgccagcaacgcggcctttttacggttcctggccttttgctggccttttgctcacatgttctttcctgcgttatcccctgattctgtggataaccgtattaccgcctttgagtgagctgataccgctcgccgcagccgaacgaccgagcgcagcgagtcagtgagcgaggaagcggaagagcgcctgatgcggtattttctccttacgcatctgtgcggtatttcacaccgcatatggtgcactctcagtacaatctgctctgatgccgcatagttaagccagtatacactccgctatcgctacgtgactgggtcatggctgcgccccgacacccgccaacacccgctgacgcgccctgacgggcttgtctgctcccggcatccgcttacagacaagctgtgaccgtctccgggagctgcatgtgtcagaggttttcaccgtcatcaccgaaacgcgcgaggcagcagatcaattcgcgcgcgaaggcgaagcggcatgcatttacgttgacaccatcgaatggtgcaaaacctttcgcggtatggcatgatagcgcccggaagagagtcaattcagggtggtgaatgtgaaaccagtaacgttatacgatgtcgcagagtatgccggtgtctcttatcagaccgtttcccgcgtggtgaaccaggccagccacgtttctgcgaaaacgcgggaaaaagtggaagcggcgatggcggagctgaattacattcccaaccgcgtggcacaacaactggcgggcaaacagtcgttgctgattggcgttgccacctccagtctggccctgcacgcgccgtcgcaaattgtcgcggcgattaaatctcgcgccgatcaactgggtgccagcgtggtggtgtcgatggtagaacgaagcggcgtcgaagcctgtaaagcggcggtgcacaatcttctcgcgcaacgcgtcagtgggctgatcattaactatccgctggatgaccaggatgccattgctgtggaagctgcctgcactaatgttccggcgttatttcttgatgtctctgaccagacacccatcaacagtattattttctcccatgaagacggtacgcgactgggcgtggagcatctggtcgcattgggtcaccagcaaatcgcgctgttagcgggcccattaagttctgtctcggcgcgtctgcgtctggctggctggcataaatatctcactcgcaatcaaattcagccgatagcggaacgggaaggcgactggagtgccatgtccggttttcaacaaaccatgcaaatgctgaatgagggcatcgttcccactgcgatgctggttgccaacgatcagatggcgctgggcgcaatgcgcgccattaccgagtccgggctgcgcgttggtgcggatatctcggtagtgggatacgacgataccgaagacagctcatgttatatcccgccgtcaaccaccatcaaacaggattttcgcctgctggggcaaaccagcgtggaccgcttgctgcaactctctcagggccaggcggtgaagggcaatcagctgttgcccgtctcactggtgaaaagaaaaaccaccctggcgcccaatacgcaaaccgcctctccccgcgcgttggccgattcattaatgcagctggcacgacaggtttcccgactggaaagcgggcagtgagcgcaacgcaattaatgtgagttagcgcgaattgatctggtttgacagcttatcatcgactgcacggtgcaccaatgcttctggcgtcaggcagccatcggaagctgtggtatggctgtgcaggtcgtaaatcactgcataattcgtgtcgctcaaggcgcactcccgttctggataatgttttttgcgccgacatcataacggttctggcaaatattctgaaatgagctgttgacaattaatcatccggctcgtataatgtgtggaattgtgagcggataacaatttcacacaggaaacagtgaacgttaaaaaggaatttcgtatgagcacgtattctgacatttgcatcgttggcgccggcataggaggcttgacttgcgccaacaacctgatcgacgccgccgccggcaggaacctgcgcatccgcgtattcgacctgaatgccaccgtaggcggccgcatccagtcgcggaaaatagatggcgaggaaatcgccgaactcggcgccgcccgctactcgccgcagctgcatccgcatttccagcaactcatgcagggcagcggcttgccgcatgcggtctacccgttcaccgaggtcatctcccacgatagcgtgctggaagagctgaaggcaacgctggatgagctgagcccgatgctgaaaatgcatccgaacgactccttcctcgagttcgtcagccattacctgggcgccgccaaggccagccacatcatcaaggcgaccggctatgacgccctgctgctgccgatggtgtcggcggccatggcctacgacatcatcaagaagcacccggaaacgcagcactttacggaaaacgccgccaaccagtggcactacgccaccgacggctaccacgaattgctgtgccagttgcagcaccaggcccaggtcgccggggtggaattcaggctcgaacaccgcttgctgtccgttgaaaaatcgggcgccgaccatgtgctcgccttcagccaccatggcgacacgcagatgcaccgcacgcgccatctggtgatggccatcccgccgtccgccatgccgcgcctgaacctggatttcccgaacgcctggagtccgttccaatacgactcgctgcccctgttcaagggattcttcacgttcgacacagcctggtgggatgcgctggggctgaccgacaaggtgctgatggcggcaaatcccctgcgcaagatctacttcaagagcgacaaatacgtgctgttctacaccgacagcaaaagcgccacctactggcgggacagcctggagcttggtgaagacgtatacctggagcgtgtccgcagccacctggaagaagtcctgccgctcgatggccagcctctgccgcagatcaaggcgcacttccacaagttctggccgcatggcgtcgagttttgcgtggagccggaagccgaccacccggccatcctgctgcaccgggacggcatcatctcctgctcggatgcctataccgcgcattgcggctggatggaaggcagcctgatcagcgcccagcacgccagcggcctgttgctgcagcgcctcgatcaacggacggaagaagaagctgccaacgataccttcatcacttcctcgaccgagcgcgcatgagcctacttgacttcccccgcctgcattttcggggttttgcccgcgccaatgtgccgacggggaatcgcaatacgcacggcaacatcgatatcgccacgaatgcggtatcgatggcgggcgaggctgtcgacctgagccggccgccagccgaattccatgcgcacctgaaacagctcgccccccgcttcaacgcacagggcaagcccgatccggacggcatcttcagccaggcgacaggctataatttttgcgggaacaaccatttctcgtgggaaaacgcgcggatcacgggcgtccagttgcgtgatggcgaggtcgatacccaggacgcgctggtgggcgccaagctgggcctgtggggccactacaacgagtacctgcgcacgacgttcaaccgcgcaaggtggatcgacaacaacccggcgcagcccgacaccacgctgatctacgcgggccagttcaccttgagcgacaagctggccacgcccaatacgcccacgctgttcacggccgacatcgcgcaggcgcactcggtgcgctggctcggcagcggccacatcacggaacgcagcgggcatttcctggacgaggaattcggccgctccaggctgttccagttttccgtggccaagcaggacccgcatttcctgttcaatccggacctgccgctgccggccagtatgcatgccttgcagcaagccctggccgacgacgaggtgctgggcctgacggtgcaatactgcctgttcaatatgtcgacgccgcaaaaacccgattcgcccgtgttctacgacctggccggcagcatcggcctgtggcggcgcggcgagctggccacctatccggccggccgcctgctgcagccgcgccagggcagcctggggccggtgctggtgaaagtgcatgcggaccgcgtctcgttcaacatgccgaccgccatccccttcaccacgcgcgacgcgggcgccgtctcggaacagcatcccacgcatgccttgggcggcaagcaggcgctgggcgacctgctgctgcatgacggcgccggcaccgttctggcgcggattcccgagcagctgtaccgcgactactggcgccatcacggcgtcttcgacgtgccgctgcagcacgctggcgcggcgccaggctcgctcagcctgggcagcgcgcaggcgcagtgggacgaagccgactgggtgctgcaatcggacagcaaccagctgtacctggaagcgccgaaccggaacaagcacgagcaatttccgcagaccatcaccgtgcaaagccgctttcgcggcgagctggcggcgcccccgtccttggcggaggcggaagacggcgtgctgctggccgtggagcagcaaccgtcgccgctcgggcacggctacacgacgctgacgctgacggggcgcaagccgggcgcgacccgcatcgtgctgggcacaggcaaggcaaagcaatacctcggcgtgcgcgtgctgcccgacgactgggacctcgacgacgtgccggccgaacaggtcgactacgccttcctctaccggcatgtgatgagctactacgagctcgtgtatcccttcatgtcggacaaggtcttcagcctggcggaccagtgcaagtgcgaaacgtattcgcgcctgatgtggcagatgtgcgatccgcagaaccgcgacaagagctactacatgcccagcacccgcgaactgtcgctgccaaagtcgcgcctgttcctgaaatacctgacgcaggtcgaggcggcagccgcggccaaggcggcggcaccggaaccggccgcgccgcatgccatcggcggcaaggcggagttgatcgacgagctgaaaaaagccatcgatctggaactgtcgctgatgctgcaatacctgtatgccgcgtattcgattcccaattatgcgcagggggcggcgctggtgcagtccggccgttggctgccggccgagctggagctggcctgcggcgccgaagaccggcgccgcaacagcggcacgcgcggcgcgctgctggaaatcgcccatgaagaaatgattcactacttattggtgaacaatgtattgatggcgcttggcgaaccgttttacagcggtaccccgctgctgggccagcaggcgcgccagcgtttcggcctggacacggaatttgcgttcgaaccattttccgaacacgtgctggcccgcttcgtgcgttttgaatggcccgactacattcccacgccgggcaaatccatcgccaccttctatatcgcgatccgccaggccctggccgagctgcccggcctgttcgaaagcggcggcggcaagcgcggcggcgagcaccacctgttcctgaaagaactgaccaaccgcgcctatcccggctaccagctggaagtatccgaccgcgacagcgcgttgttcgccatcgatttcgtcacggaacagggcgaaggcgtggccgtcgattcgccgcatttcgcctcctcgcacttccagcggctgcgcgccatcgccggcaggttttcggcctgcgacaagccgttcgaaccggcgctgccggcgctgaagaatcccgtgctggaagcgcgcgcggactgcagcgtggtgaccgatcagaaggcgcgcgcgctgatgcagctgtatcagggctgctatgaactgaccttcctgatgatggcgcaccattttgcgcagcagccgctgggcagcctgcgccgctcgcgcctgatgaacgcgtccatcgacatcatgacaggcctgttgcgccccctgtcggcggccctgatgaacatgccgtccggcctgcctggccgccatgctggaccgcccgtgcccgagccggtcagcagccgggtcagcagcgactacagcctgggctgcgacatgctggcgcagagatgcctggcgctggcgcagtacgcgcgcagcctggagagcgatgccatcggcatggcgccgatagaaatgttggagttttttaatcagcaacttaccgatttatctcggggaaagatgtcaagagaggcttgaaatgcataaaatcattatcgtcggcggaggcctggcaggcagcctcagcgccatttatctggcgcaacgggggcacgatgtccacgttgtcgaaaagcgcggcgatccgctgctggagaatgccgcaaacgccgaccccgtcaactcgcgcgccatcggcgtgagcatgacggtacgcggcatcaaggccgtcctggccgccggcatcagcaagcaggagctcgaccagtgcggcgaacccatcgtcggcatggcattcagcgtgggcggccggcaccggatacgcgagctgaccccgctcgaaggcctgttccccctgtcgctggaccgcaccgccttccagcgcctgctgaaccggcatgccgccctgcacgaggtgaagtattactttgagcataaatgcctggatgtcgacctggaaagaaagatcgtgctgatccagggcccggacggcgccttgcagaagctgcatggcgacctggtcattggcgccgacggcgcccactctgccgtgcggcgcgccatgcaaagcggcatgcgccgtttcgagttcaggcaaagttacttccgccacggctacaagacgctggtgttgccgaacgcggcggatctgggtttcaggaaggatttgctgtacttcttcggcatggattccaagggcctgtttgccggccgcgcggccaccatcccggacggcagcatcagctttgccctgtgcctgccctacaccggcacgcccagcctgggcacgctcaaccgcgaagccatggccgatttcttcagccgctacttcggcaccctgccgccggaccgtcgcaaggaaatgctggaccagttcatggcgctgcccagcaacgacctcatcaatgtccgttccagcaccttccactacaaggccaatatcctgctgatcggcgatgcggcgcatgccaccgccccgttcctcgggcaaggcatgaacatggcgctggaagacgtccacgtcttcgtgtccctgctggaaaagcacggcaatgccctgggccctgccctgtccgaattcacgcagcagcgcaaggtgcaggcggacgccatgcaggacatggcgatcgccaactatgaggcgctgagcaatccgaacctgattttcttcctgcagacgcgctacacgcgctacatgcacaagaaattcccccgtgtttatccgccagacatggcggagaaactgtacttcacatcggttccttacgatgaattgcagcaaatccagaagaaacaaaacgtttggtacaaacttggaagggtaaattaatgaaaattctcgtcatcggcgcaggccccgcaggactgctctttgccagtcaaatgaaacaggcccagcccggctgggatatcagcattacggaaaaaaacaccccggaagaagtgctgggctggggcgtggtgctgccggggcggccgccgcgccatcccgccaatccgctctcctacctggagcagtctgaacggctcaatccgcagttcctggaagaattcaagctcgtgcatcacgaccagcccaacctgatgagcaccggcgttaccctgtgcggcgtcggacgccaggccctggtgcaggcactgcgcgccaagtgcgtggcggccggcatcgccatccgttacgaaacgccgccggcggacaaggcgcagctggaagccgagtacgacctggtggtggtatcgaatggcgtcaattacaaatcgctggagttgccgccagcactggcgccacacatcgatttcggccgcaacaaatacatctggtacggcaccacccagctgttcgaccagatgaacctggtgttccgcagcaatgagcacggcatattcatcggccatgcctacaaatactcggacacgatgagcacctttatcgtcgaatgcagcgaagagacgtacgccagggccgggctggaggcgctgtccgagcgcgatgccgccgcgtacatcgccaaaacgttcaaggccgaactcggtgagcatggactgcagagccaaccgggccagggctggcgcaacttcatgaccctcagccacgaccaggcctgcgacggcaagttcgtcctgctcggcgatgcgctgcaatcggggcatttttccatcggccatggcaccaccatggcggtggtggtcgccctgctgttggtcaaaatcctcaataccgaagacggcaaggccgccgcactggacagtttcaatgcgcgtgccgtgcccctggtgcaattgttcaaggagcacgccaacaacagccgcctgtggtttgaaagcgtgggcgaacgtatcgaactgagcaatgaagagctgaccgccagcttcgacgcccgccgcaaggacttgccgtcgctacaagaagcgctgatggccagcctcggctacgcgctgggccgctaagggagataccatgccgacacacgtctccccgccgctgctgccgatgcaatggagcagcgcctatgtttcctactggacgccgatgcaggcggatgaccaggtcacctccggctattgctggttcgactatgcgcgcaatatctgccgcatcgacggcctgttcaacccctggtcggaaaaggaacatggacacctgctgtggatgtcggaaatcggcgacgccaggcgcgaacaaagccgcaagcagaaagtggcctacgcaaggcaagcggaggcggctggcgagcagctgcagggcacggcgctggccgatgaggtgaccccgttccatgagctgttcctgccgcaggcggtgctgctggacggcggtgcccgtcacgacggccgccacaccgtgctgggccgggaggcggacgcctgggtagtcgagcgggcgggcaagccgccatcggtcttttacctggaggccggtggcaaccgcctgctgcgcatggtcaccggcaatgacccgcagcacctgtcggtacgcgactttcccaacctgtttgtcagcgacattccggacagcgtctttacgtcttgcaacacctgaccggataaaacgaaaggctcagtcgaaagactgggcctttcgttttat

pEC-J-vio-2
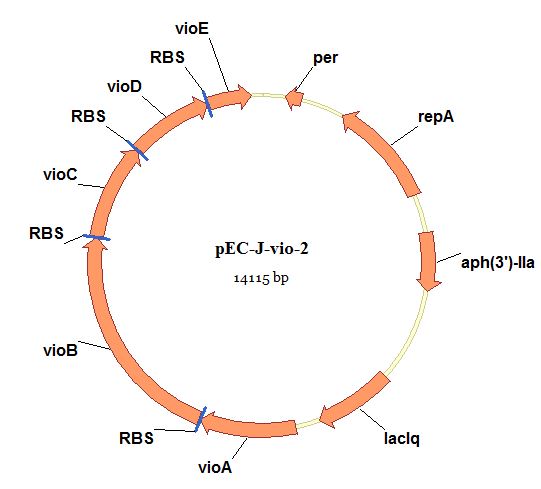


>pEC-J-vio-2

acgcccgccataaactgccaggcatcaaattaagcagaaggccatcctgacggatggcctttttgcgtttctacaaactctttttgtttatttttctaaatacattcaaatatgtatccgctcatgaattaattccgctagatgacgtgcggcttcgacctcctgggcgtggcgcttgttggcgcgctcgcggctggctgcggcacgacacgcgtctgagcagtattttgcgcgccgtcctcgtgggtcaggccggggtgggatcaggccaccgcagtaggcgcagctgatgcgatcctccactactgcgcgtcctcctggcgctgccgagcacgcagctcgtcggccagctcttcaaggtcggccacaagcgtttctaggtcgctcgcggcacttgcccagtcgcgtgatgctggcgcgtctgtcgtatcgagggcgcggaaaaatccgatcaccgtttttaaatcgacggcggcatcgagtgcgtcggactccagcgcgacatcggagagatccaccgctgatgcttcaggccagttttggtacttcgtcgtgaaggtcatgacaccattataacgaacgttcgttaaaaattctagccccaattctgataatttcttccggcactcctgcgaaaacctgcgagacttcttgcccagaaaaaacgccaagcgcagcggttaccgcactttttttccaggtgatttcaccctgaccagcgaagcggcactttagtgcatgaggtgtgcccctggtttcccctctttggagggttcaacccaaaaaagcacacaagcaaaaatgaaaatcatcatgagcaagttggtgcgaagcagcaacgcgctagcgaggaagagccagagcagaaggcggggaaccgttctctgccgacagcgtgagccccccttaaaaatcaggccggggaggaaccggggagggatcagagctaggagcgagacaccctaaagggggggaaccgttttctgctgacggtgtttcgtttattagttttcagcccgtggatagcggagggtgagggcaagtgagagccagagcaaggacgggacccctaaaggggggaaccgttttctgctgacggtgtttcgtttattagttttcagcccgtggacggccgcgtttagcttccattccaagtgcctttctgacttgttggatgcgcctttcactgacacctagttcgcctgcaagctcacgagtcgagggatcagcaaccgattgagaacgggcatccaggatcgcagttttgacgcgaagttcgagcaactcgcctgtcatttctcggcgtttgtttgcttccgctaatcgctgtcgcgtctcctgcgcatacttactttctgggtcagcccatctgcgtgcattcgatgtagctgcgccccgtcgccccatcgtcgctagagctttccgccctcggctgctctgcgtttccacccgacgagcagggacgactggctggcctttagccacgtagccgcgcacacgacgcgccatcgtcaggcgatcacgcatggcgggaagatccggctcccggccgtctgcaccgaccgcctgggcaacgttgtacgccacttcatacgcgtcgatgatcttggcatcttttaggcgctcaccagcagctttgagctggtatcccacggtcaacgcgtggcgaaacgcggtctcgtcgcgcgctcgctctggatttgtccagagcactcgcacgccgtcgatcaggtcgccggacgcgtccagggcgctcggcaggctcgcgtccaaaatcgctagcgccttggcttctgcggtggcgcgttgtgccgcttcaatgcgggcgcgtccgctggaaaagtcctgctcaatgtactttttcggcttctgtgatccggtcatcgttcgagcaatctccattaggtcggccagccgatccacacgatcatgctggcagtgccatttataggctgtcggatcgtctgagacgtgcagcggccaccggctcagcctatgcgaaaaagcctggtcagcgccgaaaacacgagtcatttcttccgtcgttgcagccagcaggcgcatatttgggctggttttacctgctgcggcatacaccgggtcaatgagccagatgagctggcatttcccgctcagcggattcacgccgatccaagccggcgctttttctaggcgtgcccatttctctaaaatcgcgtagacctgcgggtttacgtgctcaatcttcccgccggcctggtggctgggcacatcgatgtcaagcacgatcaccgcggcatgttgcgcgtgcgtcagcgcaacgtactggcaccgcgtcagcgcttttgagccagcccggtagagctttggttgggtttcgccggtatccgggtttttaatccaggcgctcgcgaaatctcttgtcttgctgccctggaagctttcgcgtcccaggtgagcgagcagttcgcggcgatcttctgccgtccagccgcgtgagccgcagcgcatagcttcggggtgggtgtcgaacagatcggcggacaatttccacgcgctagctgtgactgtgtcctgcggatcggctagagtcatgtcttgagtgctttctcccagctgatgactgggggttagccgacgccctgtgagttcccgctcacggggcgttcaactttttcaggtatttgtgcagcttatcgtgttttcttcgtaaatgaacgcttaactaccttgttaaacgtggcaaataggcaggattgatggggatctagcttcacgctgccgcaagcactcagggcgcaagggctgctaaaggaagcggaacacgtagaaagccagtccgcagaaacggtgctgaccccggatgaatgtcagctactgggctatctggacaagggaaaacgcaagcgcaaagagaaagcaggtagcttgcagtgggcttacatggcgatagctagactgggcggttttatggacagcaagcgaaccggaattgccagctggggcgccctctggtaaggttgggaagccctgcaaagtaaactggatggctttcttgccgccaaggatctgatggcgcaggggatcaagatctgatcaagagacaggatgaggatcgtttcgcatgattgaacaagatggattgcacgcaggttctccggccgcttgggtggagaggctattcggctatgactgggcacaacagacaatcggctgctctgatgccgccgtgttccggctgtcagcgcaggggcgcccggttctttttgtcaagaccgacctgtccggtgccctgaatgaactccaagacgaggcagcgcggctatcgtggctggccacgacgggcgttccttgcgcagctgtgctcgacgttgtcactgaagcgggaagggactggctgctattgggcgaagtgccggggcaggatctcctgtcatctcaccttgctcctgccgagaaagtatccatcatggctgatgcaatgcggcggctgcatacgcttgatccggctacctgcccattcgaccaccaagcgaaacatcgcatcgagcgagcacgtactcggatggaagccggtcttgtcgatcaggatgatctggacgaagagcatcaggggctcgcgccagccgaactgttcgccaggctcaaggcgcggatgcccgacggcgaggatctcgtcgtgacccatggcgatgcctgcttgccgaatatcatggtggaaaatggccgcttttctggattcatcgactgtggccggctgggtgtggcggaccgctatcaggacatagcgttggctacccgtgatattgctgaagagcttggcggcgaatgggctgaccgcttcctcgtgctttacggtatcgccgctcccgattcgcagcgcatcgccttctatcgccttcttgacgagttcttctgagcgggactctggggttcgcggaatcatgaccaaaatcccttaacgtgagttttcgttccactgagcgtcagaccccgtagaaaagatcaaaggatcttcttgagatcctttttttctgcgcgtaatctgctgcttgcaaacaaaaaaaccaccgctaccagcggtggtttgtttgccggatcaagagctaccaactctttttccgaaggtaactggcttcagcagagcgcagataccaaatactgtccttctagtgtagccgtagttaggccaccacttcaagaactctgtagcaccgcctacatacctcgctctgctaatcctgttaccagtggctgctgccagtggcgataagtcgtgtcttaccgggttggactcaagacgatagttaccggataaggcgcagcggtcgggctgaacggggggttcgtgcacacagcccagcttggagcgaacgacctacaccgaactgagatacctacagcgtgagctatgagaaagcgccacgcttcccgaagggagaaaggcggacaggtatccggtaagcggcagggtcggaacaggagagcgcacgagggagcttccagggggaaacgcctggtatctttatagtcctgtcgggtttcgccacctctgacttgagcgtcgatttttgtgatgctcgtcaggggggcggagcctatggaaaaacgccagcaacgcggcctttttacggttcctggccttttgctggccttttgctcacatgttctttcctgcgttatcccctgattctgtggataaccgtattaccgcctttgagtgagctgataccgctcgccgcagccgaacgaccgagcgcagcgagtcagtgagcgaggaagcggaagagcgcctgatgcggtattttctccttacgcatctgtgcggtatttcacaccgcatatggtgcactctcagtacaatctgctctgatgccgcatagttaagccagtatacactccgctatcgctacgtgactgggtcatggctgcgccccgacacccgccaacacccgctgacgcgccctgacgggcttgtctgctcccggcatccgcttacagacaagctgtgaccgtctccgggagctgcatgtgtcagaggttttcaccgtcatcaccgaaacgcgcgaggcagcagatcaattcgcgcgcgaaggcgaagcggcatgcatttacgttgacaccatcgaatggtgcaaaacctttcgcggtatggcatgatagcgcccggaagagagtcaattcagggtggtgaatgtgaaaccagtaacgttatacgatgtcgcagagtatgccggtgtctcttatcagaccgtttcccgcgtggtgaaccaggccagccacgtttctgcgaaaacgcgggaaaaagtggaagcggcgatggcggagctgaattacattcccaaccgcgtggcacaacaactggcgggcaaacagtcgttgctgattggcgttgccacctccagtctggccctgcacgcgccgtcgcaaattgtcgcggcgattaaatctcgcgccgatcaactgggtgccagcgtggtggtgtcgatggtagaacgaagcggcgtcgaagcctgtaaagcggcggtgcacaatcttctcgcgcaacgcgtcagtgggctgatcattaactatccgctggatgaccaggatgccattgctgtggaagctgcctgcactaatgttccggcgttatttcttgatgtctctgaccagacacccatcaacagtattattttctcccatgaagacggtacgcgactgggcgtggagcatctggtcgcattgggtcaccagcaaatcgcgctgttagcgggcccattaagttctgtctcggcgcgtctgcgtctggctggctggcataaatatctcactcgcaatcaaattcagccgatagcggaacgggaaggcgactggagtgccatgtccggttttcaacaaaccatgcaaatgctgaatgagggcatcgttcccactgcgatgctggttgccaacgatcagatggcgctgggcgcaatgcgcgccattaccgagtccgggctgcgcgttggtgcggatatctcggtagtgggatacgacgataccgaagacagctcatgttatatcccgccgtcaaccaccatcaaacaggattttcgcctgctggggcaaaccagcgtggaccgcttgctgcaactctctcagggccaggcggtgaagggcaatcagctgttgcccgtctcactggtgaaaagaaaaaccaccctggcgcccaatacgcaaaccgcctctccccgcgcgttggccgattcattaatgcagctggcacgacaggtttcccgactggaaagcgggcagtgagcgcaacgcaattaatgtgagttagcgcgaattgatctggtttgacagcttatcatcgactgcacggtgcaccaatgcttctggcgtcaggcagccatcggaagctgtggtatggctgtgcaggtcgtaaatcactgcataattcgtgtcgctcaaggcgcactcccgttctggataatgttttttgcgccgacatcataacggttctggcaaatattctgaaatgagctgttgacaattaatcatccggctcgtataatgtgtggaattgtgagcggataacaatttcacacaggaaacagtgaacgttaaaaaggaatttcgtatgagcacgtattctgacatttgcatcgttggcgccggcataggaggcttgacttgcgccaacaacctgatcgacgccgccgccggcaggaacctgcgcatccgcgtattcgacctgaatgccaccgtaggcggccgcatccagtcgcggaaaatagatggcgaggaaatcgccgaactcggcgccgcccgctactcgccgcagctgcatccgcatttccagcaactcatgcagggcagcggcttgccgcatgcggtctacccgttcaccgaggtcatctcccacgatagcgtgctggaagagctgaaggcaacgctggatgagctgagcccgatgctgaaaatgcatccgaacgactccttcctcgagttcgtcagccattacctgggcgccgccaaggccagccacatcatcaaggcgaccggctatgacgccctgctgctgccgatggtgtcggcggccatggcctacgacatcatcaagaagcacccggaaacgcagcactttacggaaaacgccgccaaccagtggcactacgccaccgacggctaccacgaattgctgtgccagttgcagcaccaggcccaggtcgccggggtggaattcaggctcgaacaccgcttgctgtccgttgaaaaatcgggcgccgaccatgtgctcgccttcagccaccatggcgacacgcagatgcaccgcacgcgccatctggtgatggccatcccgccgtccgccatgccgcgcctgaacctggatttcccgaacgcctggagtccgttccaatacgactcgctgcccctgttcaagggattcttcacgttcgacacagcctggtgggatgcgctggggctgaccgacaaggtgctgatggcggcaaatcccctgcgcaagatctacttcaagagcgacaaatacgtgctgttctacaccgacagcaaaagcgccacctactggcgggacagcctggagcttggtgaagacgtatacctggagcgtgtccgcagccacctggaagaagtcctgccgctcgatggccagcctctgccgcagatcaaggcgcacttccacaagttctggccgcatggcgtcgagttttgcgtggagccggaagccgaccacccggccatcctgctgcaccgggacggcatcatctcctgctcggatgcctataccgcgcattgcggctggatggaaggcagcctgatcagcgcccagcacgccagcggcctgttgctgcagcgcctcgatcaacggacggaagaagaagctgccaacgataccttcatcacttcctcgaccgagcgcgcatgagaaaggaggtttggacaatgagcctacttgacttcccccgcctgcattttcggggttttgcccgcgccaatgtgccgacggggaatcgcaatacgcacggcaacatcgatatcgccacgaatgcggtatcgatggcgggcgaggctgtcgacctgagccggccgccagccgaattccatgcgcacctgaaacagctcgccccccgcttcaacgcacagggcaagcccgatccggacggcatcttcagccaggcgacaggctataatttttgcgggaacaaccatttctcgtgggaaaacgcgcggatcacgggcgtccagttgcgtgatggcgaggtcgatacccaggacgcgctggtgggcgccaagctgggcctgtggggccactacaacgagtacctgcgcacgacgttcaaccgcgcaaggtggatcgacaacaacccggcgcagcccgacaccacgctgatctacgcgggccagttcaccttgagcgacaagctggccacgcccaatacgcccacgctgttcacggccgacatcgcgcaggcgcactcggtgcgctggctcggcagcggccacatcacggaacgcagcgggcatttcctggacgaggaattcggccgctccaggctgttccagttttccgtggccaagcaggacccgcatttcctgttcaatccggacctgccgctgccggccagtatgcatgccttgcagcaagccctggccgacgacgaggtgctgggcctgacggtgcaatactgcctgttcaatatgtcgacgccgcaaaaacccgattcgcccgtgttctacgacctggccggcagcatcggcctgtggcggcgcggcgagctggccacctatccggccggccgcctgctgcagccgcgccagggcagcctggggccggtgctggtgaaagtgcatgcggaccgcgtctcgttcaacatgccgaccgccatccccttcaccacgcgcgacgcgggcgccgtctcggaacagcatcccacgcatgccttgggcggcaagcaggcgctgggcgacctgctgctgcatgacggcgccggcaccgttctggcgcggattcccgagcagctgtaccgcgactactggcgccatcacggcgtcttcgacgtgccgctgcagcacgctggcgcggcgccaggctcgctcagcctgggcagcgcgcaggcgcagtgggacgaagccgactgggtgctgcaatcggacagcaaccagctgtacctggaagcgccgaaccggaacaagcacgagcaatttccgcagaccatcaccgtgcaaagccgctttcgcggcgagctggcggcgcccccgtccttggcggaggcggaagacggcgtgctgctggccgtggagcagcaaccgtcgccgctcgggcacggctacacgacgctgacgctgacggggcgcaagccgggcgcgacccgcatcgtgctgggcacaggcaaggcaaagcaatacctcggcgtgcgcgtgctgcccgacgactgggacctcgacgacgtgccggccgaacaggtcgactacgccttcctctaccggcatgtgatgagctactacgagctcgtgtatcccttcatgtcggacaaggtcttcagcctggcggaccagtgcaagtgcgaaacgtattcgcgcctgatgtggcagatgtgcgatccgcagaaccgcgacaagagctactacatgcccagcacccgcgaactgtcgctgccaaagtcgcgcctgttcctgaaatacctgacgcaggtcgaggcggcagccgcggccaaggcggcggcaccggaaccggccgcgccgcatgccatcggcggcaaggcggagttgatcgacgagctgaaaaaagccatcgatctggaactgtcgctgatgctgcaatacctgtatgccgcgtattcgattcccaattatgcgcagggggcggcgctggtgcagtccggccgttggctgccggccgagctggagctggcctgcggcgccgaagaccggcgccgcaacagcggcacgcgcggcgcgctgctggaaatcgcccatgaagaaatgattcactacttattggtgaacaatgtattgatggcgcttggcgaaccgttttacagcggtaccccgctgctgggccagcaggcgcgccagcgtttcggcctggacacggaatttgcgttcgaaccattttccgaacacgtgctggcccgcttcgtgcgttttgaatggcccgactacattcccacgccgggcaaatccatcgccaccttctatatcgcgatccgccaggccctggccgagctgcccggcctgttcgaaagcggcggcggcaagcgcggcggcgagcaccacctgttcctgaaagaactgaccaaccgcgcctatcccggctaccagctggaagtatccgaccgcgacagcgcgttgttcgccatcgatttcgtcacggaacagggcgaaggcgtggccgtcgattcgccgcatttcgcctcctcgcacttccagcggctgcgcgccatcgccggcaggttttcggcctgcgacaagccgttcgaaccggcgctgccggcgctgaagaatcccgtgctggaagcgcgcgcggactgcagcgtggtgaccgatcagaaggcgcgcgcgctgatgcagctgtatcagggctgctatgaactgaccttcctgatgatggcgcaccattttgcgcagcagccgctgggcagcctgcgccgctcgcgcctgatgaacgcgtccatcgacatcatgacaggcctgttgcgccccctgtcggcggccctgatgaacatgccgtccggcctgcctggccgccatgctggaccgcccgtgcccgagccggtcagcagccgggtcagcagcgactacagcctgggctgcgacatgctggcgcagagatgcctggcgctggcgcagtacgcgcgcagcctggagagcgatgccatcggcatggcgccgatagaaatgttggagttttttaatcagcaacttaccgatttatctcggggaaagatgtcaagagaggcttgagaaaggaggtttggacaatgcataaaatcattatcgtcggcggaggcctggcaggcagcctcagcgccatttatctggcgcaacgggggcacgatgtccacgttgtcgaaaagcgcggcgatccgctgctggagaatgccgcaaacgccgaccccgtcaactcgcgcgccatcggcgtgagcatgacggtacgcggcatcaaggccgtcctggccgccggcatcagcaagcaggagctcgaccagtgcggcgaacccatcgtcggcatggcattcagcgtgggcggccggcaccggatacgcgagctgaccccgctcgaaggcctgttccccctgtcgctggaccgcaccgccttccagcgcctgctgaaccggcatgccgccctgcacgaggtgaagtattactttgagcataaatgcctggatgtcgacctggaaagaaagatcgtgctgatccagggcccggacggcgccttgcagaagctgcatggcgacctggtcattggcgccgacggcgcccactctgccgtgcggcgcgccatgcaaagcggcatgcgccgtttcgagttcaggcaaagttacttccgccacggctacaagacgctggtgttgccgaacgcggcggatctgggtttcaggaaggatttgctgtacttcttcggcatggattccaagggcctgtttgccggccgcgcggccaccatcccggacggcagcatcagctttgccctgtgcctgccctacaccggcacgcccagcctgggcacgctcaaccgcgaagccatggccgatttcttcagccgctacttcggcaccctgccgccggaccgtcgcaaggaaatgctggaccagttcatggcgctgcccagcaacgacctcatcaatgtccgttccagcaccttccactacaaggccaatatcctgctgatcggcgatgcggcgcatgccaccgccccgttcctcgggcaaggcatgaacatggcgctggaagacgtccacgtcttcgtgtccctgctggaaaagcacggcaatgccctgggccctgccctgtccgaattcacgcagcagcgcaaggtgcaggcggacgccatgcaggacatggcgatcgccaactatgaggcgctgagcaatccgaacctgattttcttcctgcagacgcgctacacgcgctacatgcacaagaaattcccccgtgtttatccgccagacatggcggagaaactgtacttcacatcggttccttacgatgaattgcagcaaatccagaagaaacaaaacgtttggtacaaacttggaagggtaaattaagaaaggaggtttggacaatgaaaattctcgtcatcggcgcaggccccgcaggactgctctttgccagtcaaatgaaacaggcccagcccggctgggatatcagcattacggaaaaaaacaccccggaagaagtgctgggctggggcgtggtgctgccggggcggccgccgcgccatcccgccaatccgctctcctacctggagcagtctgaacggctcaatccgcagttcctggaagaattcaagctcgtgcatcacgaccagcccaacctgatgagcaccggcgttaccctgtgcggcgtcggacgccaggccctggtgcaggcactgcgcgccaagtgcgtggcggccggcatcgccatccgttacgaaacgccgccggcggacaaggcgcagctggaagccgagtacgacctggtggtggtatcgaatggcgtcaattacaaatcgctggagttgccgccagcactggcgccacacatcgatttcggccgcaacaaatacatctggtacggcaccacccagctgttcgaccagatgaacctggtgttccgcagcaatgagcacggcatattcatcggccatgcctacaaatactcggacacgatgagcacctttatcgtcgaatgcagcgaagagacgtacgccagggccgggctggaggcgctgtccgagcgcgatgccgccgcgtacatcgccaaaacgttcaaggccgaactcggtgagcatggactgcagagccaaccgggccagggctggcgcaacttcatgaccctcagccacgaccaggcctgcgacggcaagttcgtcctgctcggcgatgcgctgcaatcggggcatttttccatcggccatggcaccaccatggcggtggtggtcgccctgctgttggtcaaaatcctcaataccgaagacggcaaggccgccgcactggacagtttcaatgcgcgtgccgtgcccctggtgcaattgttcaaggagcacgccaacaacagccgcctgtggtttgaaagcgtgggcgaacgtatcgaactgagcaatgaagagctgaccgccagcttcgacgcccgccgcaaggacttgccgtcgctacaagaagcgctgatggccagcctcggctacgcgctgggccgctaagaaaggaggtttggacaatgccgacacacgtctccccgccgctgctgccgatgcaatggagcagcgcctatgtttcctactggacgccgatgcaggcggatgaccaggtcacctccggctattgctggttcgactatgcgcgcaatatctgccgcatcgacggcctgttcaacccctggtcggaaaaggaacatggacacctgctgtggatgtcggaaatcggcgacgccaggcgcgaacaaagccgcaagcagaaagtggcctacgcaaggcaagcggaggcggctggcgagcagctgcagggcacggcgctggccgatgaggtgaccccgttccatgagctgttcctgccgcaggcggtgctgctggacggcggtgcccgtcacgacggccgccacaccgtgctgggccgggaggcggacgcctgggtagtcgagcgggcgggcaagccgccatcggtcttttacctggaggccggtggcaaccgcctgctgcgcatggtcaccggcaatgacccgcagcacctgtcggtacgcgactttcccaacctgtttgtcagcgacattccggacagcgtctttacgtcttgcaacacctgaccggataaaacgaaaggctcagtcgaaagactgggcctttcgttttatctgttgtttgtcggtgaacgctctcctgagtaggacaaatccgccgggagcggatttgaacgttgcgaagcaacggcccggagggtggcgggcagg

pEC-C-vio-1


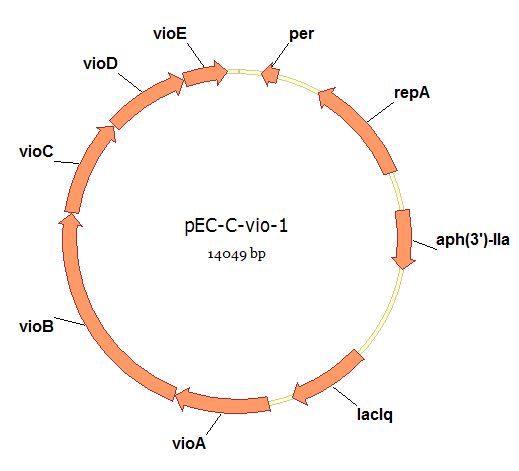


>pEC-C-vio-1

gggcaggacgcccgccataaactgccaggcatcaaattaagcagaaggccatcctgacggatggcctttttgcgtttctacaaactctttttgtttatttttctaaatacattcaaatatgtatccgctcatgaattaattccgctagatgacgtgcggcttcgacctcctgggcgtggcgcttgttggcgcgctcgcggctggctgcggcacgacacgcgtctgagcagtattttgcgcgccgtcctcgtgggtcaggccggggtgggatcaggccaccgcagtaggcgcagctgatgcgatcctccactactgcgcgtcctcctggcgctgccgagcacgcagctcgtcggccagctcttcaaggtcggccacaagcgtttctaggtcgctcgcggcacttgcccagtcgcgtgatgctggcgcgtctgtcgtatcgagggcgcggaaaaatccgatcaccgtttttaaatcgacggcggcatcgagtgcgtcggactccagcgcgacatcggagagatccaccgctgatgcttcaggccagttttggtacttcgtcgtgaaggtcatgacaccattataacgaacgttcgttaaaaattctagccccaattctgataatttcttccggcactcctgcgaaaacctgcgagacttcttgcccagaaaaaacgccaagcgcagcggttaccgcactttttttccaggtgatttcaccctgaccagcgaagcggcactttagtgcatgaggtgtgcccctggtttcccctctttggagggttcaacccaaaaaagcacacaagcaaaaatgaaaatcatcatgagcaagttggtgcgaagcagcaacgcgctagcgaggaagagccagagcagaaggcggggaaccgttctctgccgacagcgtgagccccccttaaaaatcaggccggggaggaaccggggagggatcagagctaggagcgagacaccctaaagggggggaaccgttttctgctgacggtgtttcgtttattagttttcagcccgtggatagcggagggtgagggcaagtgagagccagagcaaggacgggacccctaaaggggggaaccgttttctgctgacggtgtttcgtttattagttttcagcccgtggacggccgcgtttagcttccattccaagtgcctttctgacttgttggatgcgcctttcactgacacctagttcgcctgcaagctcacgagtcgagggatcagcaaccgattgagaacgggcatccaggatcgcagttttgacgcgaagttcgagcaactcgcctgtcatttctcggcgtttgtttgcttccgctaatcgctgtcgcgtctcctgcgcatacttactttctgggtcagcccatctgcgtgcattcgatgtagctgcgccccgtcgccccatcgtcgctagagctttccgccctcggctgctctgcgtttccacccgacgagcagggacgactggctggcctttagccacgtagccgcgcacacgacgcgccatcgtcaggcgatcacgcatggcgggaagatccggctcccggccgtctgcaccgaccgcctgggcaacgttgtacgccacttcatacgcgtcgatgatcttggcatcttttaggcgctcaccagcagctttgagctggtatcccacggtcaacgcgtggcgaaacgcggtctcgtcgcgcgctcgctctggatttgtccagagcactcgcacgccgtcgatcaggtcgccggacgcgtccagggcgctcggcaggctcgcgtccaaaatcgctagcgccttggcttctgcggtggcgcgttgtgccgcttcaatgcgggcgcgtccgctggaaaagtcctgctcaatgtactttttcggcttctgtgatccggtcatcgttcgagcaatctccattaggtcggccagccgatccacacgatcatgctggcagtgccatttataggctgtcggatcgtctgagacgtgcagcggccaccggctcagcctatgcgaaaaagcctggtcagcgccgaaaacacgagtcatttcttccgtcgttgcagccagcaggcgcatatttgggctggttttacctgctgcggcatacaccgggtcaatgagccagatgagctggcatttcccgctcagcggattcacgccgatccaagccggcgctttttctaggcgtgcccatttctctaaaatcgcgtagacctgcgggtttacgtgctcaatcttcccgccggcctggtggctgggcacatcgatgtcaagcacgatcaccgcggcatgttgcgcgtgcgtcagcgcaacgtactggcaccgcgtcagcgcttttgagccagcccggtagagctttggttgggtttcgccggtatccgggtttttaatccaggcgctcgcgaaatctcttgtcttgctgccctggaagctttcgcgtcccaggtgagcgagcagttcgcggcgatcttctgccgtccagccgcgtgagccgcagcgcatagcttcggggtgggtgtcgaacagatcggcggacaatttccacgcgctagctgtgactgtgtcctgcggatcggctagagtcatgtcttgagtgctttctcccagctgatgactgggggttagccgacgccctgtgagttcccgctcacggggcgttcaactttttcaggtatttgtgcagcttatcgtgttttcttcgtaaatgaacgcttaactaccttgttaaacgtggcaaataggcaggattgatggggatctagcttcacgctgccgcaagcactcagggcgcaagggctgctaaaggaagcggaacacgtagaaagccagtccgcagaaacggtgctgaccccggatgaatgtcagctactgggctatctggacaagggaaaacgcaagcgcaaagagaaagcaggtagcttgcagtgggcttacatggcgatagctagactgggcggttttatggacagcaagcgaaccggaattgccagctggggcgccctctggtaaggttgggaagccctgcaaagtaaactggatggctttcttgccgccaaggatctgatggcgcaggggatcaagatctgatcaagagacaggatgaggatcgtttcgcatgattgaacaagatggattgcacgcaggttctccggccgcttgggtggagaggctattcggctatgactgggcacaacagacaatcggctgctctgatgccgccgtgttccggctgtcagcgcaggggcgcccggttctttttgtcaagaccgacctgtccggtgccctgaatgaactccaagacgaggcagcgcggctatcgtggctggccacgacgggcgttccttgcgcagctgtgctcgacgttgtcactgaagcgggaagggactggctgctattgggcgaagtgccggggcaggatctcctgtcatctcaccttgctcctgccgagaaagtatccatcatggctgatgcaatgcggcggctgcatacgcttgatccggctacctgcccattcgaccaccaagcgaaacatcgcatcgagcgagcacgtactcggatggaagccggtcttgtcgatcaggatgatctggacgaagagcatcaggggctcgcgccagccgaactgttcgccaggctcaaggcgcggatgcccgacggcgaggatctcgtcgtgacccatggcgatgcctgcttgccgaatatcatggtggaaaatggccgcttttctggattcatcgactgtggccggctgggtgtggcggaccgctatcaggacatagcgttggctacccgtgatattgctgaagagcttggcggcgaatgggctgaccgcttcctcgtgctttacggtatcgccgctcccgattcgcagcgcatcgccttctatcgccttcttgacgagttcttctgagcgggactctggggttcgcggaatcatgaccaaaatcccttaacgtgagttttcgttccactgagcgtcagaccccgtagaaaagatcaaaggatcttcttgagatcctttttttctgcgcgtaatctgctgcttgcaaacaaaaaaaccaccgctaccagcggtggtttgtttgccggatcaagagctaccaactctttttccgaaggtaactggcttcagcagagcgcagataccaaatactgtccttctagtgtagccgtagttaggccaccacttcaagaactctgtagcaccgcctacatacctcgctctgctaatcctgttaccagtggctgctgccagtggcgataagtcgtgtcttaccgggttggactcaagacgatagttaccggataaggcgcagcggtcgggctgaacggggggttcgtgcacacagcccagcttggagcgaacgacctacaccgaactgagatacctacagcgtgagctatgagaaagcgccacgcttcccgaagggagaaaggcggacaggtatccggtaagcggcagggtcggaacaggagagcgcacgagggagcttccagggggaaacgcctggtatctttatagtcctgtcgggtttcgccacctctgacttgagcgtcgatttttgtgatgctcgtcaggggggcggagcctatggaaaaacgccagcaacgcggcctttttacggttcctggccttttgctggccttttgctcacatgttctttcctgcgttatcccctgattctgtggataaccgtattaccgcctttgagtgagctgataccgctcgccgcagccgaacgaccgagcgcagcgagtcagtgagcgaggaagcggaagagcgcctgatgcggtattttctccttacgcatctgtgcggtatttcacaccgcatatggtgcactctcagtacaatctgctctgatgccgcatagttaagccagtatacactccgctatcgctacgtgactgggtcatggctgcgccccgacacccgccaacacccgctgacgcgccctgacgggcttgtctgctcccggcatccgcttacagacaagctgtgaccgtctccgggagctgcatgtgtcagaggttttcaccgtcatcaccgaaacgcgcgaggcagcagatcaattcgcgcgcgaaggcgaagcggcatgcatttacgttgacaccatcgaatggtgcaaaacctttcgcggtatggcatgatagcgcccggaagagagtcaattcagggtggtgaatgtgaaaccagtaacgttatacgatgtcgcagagtatgccggtgtctcttatcagaccgtttcccgcgtggtgaaccaggccagccacgtttctgcgaaaacgcgggaaaaagtggaagcggcgatggcggagctgaattacattcccaaccgcgtggcacaacaactggcgggcaaacagtcgttgctgattggcgttgccacctccagtctggccctgcacgcgccgtcgcaaattgtcgcggcgattaaatctcgcgccgatcaactgggtgccagcgtggtggtgtcgatggtagaacgaagcggcgtcgaagcctgtaaagcggcggtgcacaatcttctcgcgcaacgcgtcagtgggctgatcattaactatccgctggatgaccaggatgccattgctgtggaagctgcctgcactaatgttccggcgttatttcttgatgtctctgaccagacacccatcaacagtattattttctcccatgaagacggtacgcgactgggcgtggagcatctggtcgcattgggtcaccagcaaatcgcgctgttagcgggcccattaagttctgtctcggcgcgtctgcgtctggctggctggcataaatatctcactcgcaatcaaattcagccgatagcggaacgggaaggcgactggagtgccatgtccggttttcaacaaaccatgcaaatgctgaatgagggcatcgttcccactgcgatgctggttgccaacgatcagatggcgctgggcgcaatgcgcgccattaccgagtccgggctgcgcgttggtgcggatatctcggtagtgggatacgacgataccgaagacagctcatgttatatcccgccgtcaaccaccatcaaacaggattttcgcctgctggggcaaaccagcgtggaccgcttgctgcaactctctcagggccaggcggtgaagggcaatcagctgttgcccgtctcactggtgaaaagaaaaaccaccctggcgcccaatacgcaaaccgcctctccccgcgcgttggccgattcattaatgcagctggcacgacaggtttcccgactggaaagcgggcagtgagcgcaacgcaattaatgtgagttagcgcgaattgatctggtttgacagcttatcatcgactgcacggtgcaccaatgcttctggcgtcaggcagccatcggaagctgtggtatggctgtgcaggtcgtaaatcactgcataattcgtgtcgctcaaggcgcactcccgttctggataatgttttttgcgccgacatcataacggttctggcaaatattctgaaatgagctgttgacaattaatcatccggctcgtataatgtgtggaattgtgagcggataacaatttcacacaggaaacagtgaacgttaaaaaggaatttcgtatgaagcactcttctgacatctgtatcgttggtgctggtatctctggtttgacctgtgcttctcacttgttggactctccagcttgtagaggtttgtctttgagaatcttcgacatgcaacaagaagctggtggtagaatccgatctaagatgttggacggtaaggcttctatcgaattgggtgctggtagatactctccacaattgcacccacacttccaatctgctatgcaacactactctcaaaagtctgaagtttacccattcacccaattgaagttcaagtctcacgttcaacaaaagttgaagagagctatgaacgaattgtctccaagattgaaggaacacggtaaggaatctttcttgcaattcgttagtagataccaaggtcacgactctgctgttggtatgatccgatctatgggttacgacgctttgttcttgccagacatctctgctgaaatggcttacgacatcgttggtaagcacccagaaatccaatctgttaccgacaacgacgctaaccaatggttcgctgctgaaacaggtttcgctggtttgatccaaggtatcaaggctaaggttaaggctgctggtgctagattctctttgggttacagattgttgtctgttagaaccgacggtgacggttacttgttgcaattggctggtgacgacggttggaagttggaacacagaaccagacacttgatcttggctatcccaccatctgctatggctggtttgaacgttgacttcccagaggcttggtctggtgctagatacggttctttgccattgttcaagggtttcttgacctacggtgaaccatggtggttggactacaagttggacgaccaagttttgatcgttgacaacccattgagaaaaatctacttcaagggtgacaagtacttgttcttctacaccgactctgaaatggctaactactggagaggttgtgttgctgaaggtgaagacggttacttggaacaaatcagaacccacttggcttctgctttgggtatcgttagagaaagaatcccacaaccattggctcacgttcacaagtactgggctcacggtgttgagttctgtagagactctgacatcgaccacccatctgctttgtctcacagagactctggtatcatcgcttgttctgacgcttacaccgaacactgtggttggatggaaggtggtttgttgtctgctagagaggctagtagattgttgttgcaaagaatcgctgctttgagaagataggaaaggaggtttggacaatgagcattctggatttcccgcgtatccacttccgtggctgggcccgtgtcaatgcgccgaccgcgaaccgcgatccgcacggccacatcgatatggccagcaataccgtggcgatggcgggtgagccgttcgacctggcacgccatcctacggagttccaccgtcacctgcgctccctgggtccgcgcttcggcttggatggtcgtgctgacccggaaggcccgttcagcctggccgagggctacaacgctgccggtaacaaccacttttcgtgggagagcgcaaccgttagccacgtgcaatgggatggcggtgaggcggatcgtggtgacggtctggtcggtgctcgtttggcactgtggggtcactacaatgattatctgcgtaccaccttcaatcgtgctcgttgggtcgacagcgacccgacgcgccgtgacgctgcacaaatctatgcgggccaattcaccattagcccggctggtgccggtccgggtacgccgtggctgtttacggcagacattgatgatagccatggtgcacgttggacgcgtggcggccacattgcagagcgtggcggccacttcttggatgaagagtttggtctggcacgcctgtttcagttctctgtgccgaaagatcacccacattttctgtttcacccgggtccgtttgattccgaggcctggcgtcgtctgcaattggctctggaggatgacgacgttctgggtctgaccgtgcaatatgcgttgttcaatatgagcaccccgcctcagccgaacagcccggtttttcacgatatggtcggtgttgtcggtctgtggcgtcgtggtgaactggcgagctacccggctggtcgtctgctgcgtccgcgtcaaccgggtctgggtgacctgaccctgcgcgtcaacggtggtcgcgttgcgctgaatttggcgtgtgccattccgttcagcactcgtgccgcgcagccaagcgcaccggaccgcctgaccccggacctgggtgccaaactgccgctgggcgatctgctgctgcgtgatgaggacggcgcactgttggcacgtgtgccgcaggctctgtaccaagactattggacgaatcacggtattgtggacctgccgctgctgcgcgaaccgcgtggtagcttgaccctgagcagcgaactggcggagtggcgtgagcaagactgggtcacccaaagcgacgcgtctaacctgtacctggaggcaccggatcgccgtcacggtcgctttttccctgagagcatcgcgctgcgcagctactttcgcggtgaagcgcgtgcgcgtccggatatcccgcatcgtatcgagggcatgggcctggtcggcgtcgaatctcgtcaggatggcgacgctgcggaatggcgtctgacgggtctgcgtccgggtccggcacgcattgttctggacgatggtgccgaggcgatccctctgcgtgttctgcctgacgattgggcgctggatgacgcgaccgtcgaagaagtggattacgcctttttgtaccgccacgttatggcgtattacgagctggtgtatccattcatgagcgacaaggtgttttccctggctgatcgttgcaaatgtgaaacgtacgcacgtctgatgtggcagatgtgtgatccgcagaaccgcaacaagtcctattacatgccgagcacccgcgaactgtcggcaccgaaagctcgtttgttcttgaagtatctggcccacgtggaaggccaggcacgcctgcaagcacctccgccagcgggtccggcacgcattgaatctaaagcccagttggcggcagagctgcgtaaagccgtcgacctggagctgtctgtgatgctgcaatacctgtacgcggcgtatagcattccgaactatgcacagggccaacaacgtgttcgtgacggtgcgtggaccgccgagcagctgcaactggcgtgcggtagcggtgaccgtcgccgtgatggcggtattcgtgcagcactgctggaaattgctcatgaagaaatgattcattacctggtcgttaacaacctgctgatggccctgggcgagccgttctacgcgggtgtcccgctgatgggcgaagcggcacgtcaggcgtttggcctggacaccgagttcgctctggaaccgtttagcgaaagcacgctggcacgttttgttcgtctggaatggccgcactttatcccagcaccgggcaaatccatcgcggactgctatgccgccattcgtcaggcgtttttggatctgccggacttgtttggtggcgaggcaggtaagcgtggcggtgaacaccacctgttcctgaatgagctgaccaaccgtgcgcatccgggttatcaactggaagttttcgatcgcgactcggcgctgtttggtattgcatttgtgaccgatcagggcgaaggtggcgctctggacagcccgcactacgaacatagccattttcaacgtctgcgtgaaatgagcgcgcgtatcatggctcaaagcgcaccgttcgaaccggcgctgccggcgttgcgtaatccggttctggatgagagcccgggttgccaacgtgtcgcagacggtcgtgcgcgtgcgctgatggcattgtaccaaggcgtttatgagctgatgtttgcgatgatggcgcagcacttcgccgtgaaaccgctgggtagcttgcgtcgcagccgcctgatgaacgcagcaatcgatctgatgaccggtctgttgcgtccgctgagctgcgcgctgatgaacctgccaagcggcatcgccggtcgcacggccggtccgccgctgccgggtccggttgacacccgtagctatgacgactacgcgctgggctgtcgcatgctggcacgccgttgcgagcgtctgctggagcaggcgagcatgctggaaccgggttggctgccggatgcgcagatggagctgctggatttctatcgtcgccaaatgctggacttggcgtgcggcaaactgagccgcgaggcctaataggaaaggaggtttggacaatgaaaagagctatcatcgttggtggtggtttggctggtggtttgaccgctatctacttggctaagagaggttacgaagttcacgttgttgaaaagagaggtgacccattgagagacttgtcttcttacgttgacgttgtttctagtagagctatcggtgtttctatgaccgttagaggtatcaagtctgttttggctgctggtatcccaagagctgaattggacgcttgtggtgaaccaatcgttgctatggctttctctgttggtggtcaatacagaatgagagaattgaagccattggaagacttcagaccattgtctttgaacagagctgctttccaaaagttgttgaacaagtacgctaacttggctggtgttagatactacttcgaacacaagtgtttggacgttgacttggacggtaagtctgttttgatccaaggtaaggacggtcaaccacaaagattgcaaggtgacatgatcatcggtgctgacggtgctcactctgctgttagacaagctatgcaatctggtttgagaagattcgagttccaacaaaccttcttcagacacggttacaagaccttggttttgccagacgctcaggctttgggttacagaaaggacaccttgtacttcttcggtatggactctggtggtttgttcgctggtagagctgctaccatcccagacggttctgtttctatcgctgtttgtttgccatactctggttctccatctttgaccaccaccgacgaaccaaccatgagagctttcttcgacagatacttcggtggtttgccacgcgacgctcgcgacgaaatgttgagacaattcttggctaagccatctaacgacttgatcaacgttcgatcttctaccttccactacaagggtaacgttttgttgttgggtgacgctgctcacgctaccgctccattcttgggtcaaggtatgaacatggctttggaagacgctagaaccttcgttgaattgttggacagacaccaaggtgaccaagacaaggctttcccagagttcaccgaattgagaaaggttcaagctgacgctatgcaagacatggctagagctaactacgacgttttgtcttgttctaacccaatcttcttcatgagagctagatacaccagatacatgcactctaagttcccaggtttgtacccaccagacatggctgaaaagttgtacttcacctctgaaccatacgacagattgcaacaaatccaaagaaagcaaaacgtttggtacaagatcggtagagttaactaggaaaggaggtttggacaatgaaaatcttggttatcggtgctggtccagctggtttggttttcgcttctcaattgaagcaagctagaccattgtgggctatcgacatcgttgaaaagaacgacgaacaagaagttttgggttggggtgttgttttgccaggtagaccaggtcaacacccagctaacccattgtcttacttggacgctccagaaagattgaacccacaattcttggaagacttcaagttggttcaccacaacgaaccatctttgatgtctaccggtgttttgttgtgtggtgttgaaagaagaggtttggttcacgctttgagagacaagtgtcgatctcaaggtatcgctatcagattcgaatctccattgttggaacacggtgaattgccattggctgactacgacttggttgttttggctaacggtgttaaccacaagaccgctcacttcaccgaggctttggttccacaagttgactacggtagaaacaagtacatctggtacggaacctctcaattgttcgaccaaatgaacttggttttcagaacccacggtaaggacatcttcatcgctcacgcttacaagtactctgacaccatgtctaccttcatcgttgaatgttctgaagaaacctacgctagagctagattgggtgaaatgtctgaagaggcttctgctgaatacgttgctaaggttttccaagctgaattgggtggtcacggtttggtttctcaaccaggtttgggttggagaaacttcatgaccttgtctcacgacagatgtcacgacggtaagttggttttgttgggtgacgctttgcaatctggtcacttctctatcggtcacggaaccacaatggctgttgttgttgctcaattgttggttaaggctttgtgtaccgaagacggtgttccagctgctttgaagagattcgaagaaagagctttgccattggttcaattgttcagaggtcacgctgacaacagtagagtttggttcgaaaccgttgaagaaagaatgcacttgtcttctgctgagttcgttcaatctttcgacgctagaagaaagtctttgccaccaatgccagaggctttggctcaaaacttgagatacgctttgcaaagataggaaaggaggtttggacaatggaaaacagagaaccaccattgttgccagctagatggtcttctgcttacgtttcttactggagtccaatgttgccagacgaccaattgacctctggttactgttggttcgactacgaaagagacatctgtagaatcgacggtttgttcaacccgtggtctgaaagagacaccggttacagattgtggatgtctgaagttggtaacgctgcttctggtagaacctggaagcaaaaggttgcttacggtagagaaagaaccgctttgggtgaacaattgtgtgaaagaccattggacgacgaaaccggtccattcgctgaattgttcttgccacgcgacgttttgagaagattgggtgctagacacatcggtagaagagttgttttgggtagagaagctgacggttggagataccaaagaccaggtaagggtccatctaccttgtacttggacgctgcttctggtaccccattgagaatggttaccggtgacgaggctagtagagcttctttgagagacttcccaaacgtttctgaagctgaaatcccagacgctgttttcgctgctaagagatagccggataaaacgaaaggctcagtcgaaagactgggcctttcgttttatctgttgtttgtcggtgaacgctctcctgagtaggacaaatccgccgggagcggatttgaacgttgcgaagcaacggcccggagggtggc

**pEC-C-vio-2**


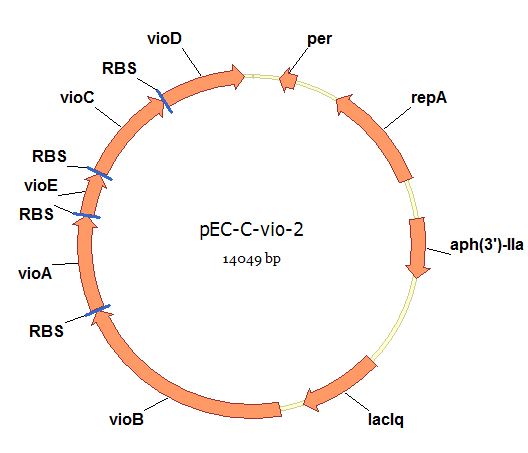


**>pEC-C-vio-2**

ggagcggatttgaacgttgcgaagcaacggcccggagggtggcgggcaggacgcccgccataaactgccaggcatcaaattaagcagaaggccatcctgacggatggcctttttgcgtttctacaaactctttttgtttatttttctaaatacattcaaatatgtatccgctcatgaattaattccgctagatgacgtgcggcttcgacctcctgggcgtggcgcttgttggcgcgctcgcggctggctgcggcacgacacgcgtctgagcagtattttgcgcgccgtcctcgtgggtcaggccggggtgggatcaggccaccgcagtaggcgcagctgatgcgatcctccactactgcgcgtcctcctggcgctgccgagcacgcagctcgtcggccagctcttcaaggtcggccacaagcgtttctaggtcgctcgcggcacttgcccagtcgcgtgatgctggcgcgtctgtcgtatcgagggcgcggaaaaatccgatcaccgtttttaaatcgacggcggcatcgagtgcgtcggactccagcgcgacatcggagagatccaccgctgatgcttcaggccagttttggtacttcgtcgtgaaggtcatgacaccattataacgaacgttcgttaaaaattctagccccaattctgataatttcttccggcactcctgcgaaaacctgcgagacttcttgcccagaaaaaacgccaagcgcagcggttaccgcactttttttccaggtgatttcaccctgaccagcgaagcggcactttagtgcatgaggtgtgcccctggtttcccctctttggagggttcaacccaaaaaagcacacaagcaaaaatgaaaatcatcatgagcaagttggtgcgaagcagcaacgcgctagcgaggaagagccagagcagaaggcggggaaccgttctctgccgacagcgtgagccccccttaaaaatcaggccggggaggaaccggggagggatcagagctaggagcgagacaccctaaagggggggaaccgttttctgctgacggtgtttcgtttattagttttcagcccgtggatagcggagggtgagggcaagtgagagccagagcaaggacgggacccctaaaggggggaaccgttttctgctgacggtgtttcgtttattagttttcagcccgtggacggccgcgtttagcttccattccaagtgcctttctgacttgttggatgcgcctttcactgacacctagttcgcctgcaagctcacgagtcgagggatcagcaaccgattgagaacgggcatccaggatcgcagttttgacgcgaagttcgagcaactcgcctgtcatttctcggcgtttgtttgcttccgctaatcgctgtcgcgtctcctgcgcatacttactttctgggtcagcccatctgcgtgcattcgatgtagctgcgccccgtcgccccatcgtcgctagagctttccgccctcggctgctctgcgtttccacccgacgagcagggacgactggctggcctttagccacgtagccgcgcacacgacgcgccatcgtcaggcgatcacgcatggcgggaagatccggctcccggccgtctgcaccgaccgcctgggcaacgttgtacgccacttcatacgcgtcgatgatcttggcatcttttaggcgctcaccagcagctttgagctggtatcccacggtcaacgcgtggcgaaacgcggtctcgtcgcgcgctcgctctggatttgtccagagcactcgcacgccgtcgatcaggtcgccggacgcgtccagggcgctcggcaggctcgcgtccaaaatcgctagcgccttggcttctgcggtggcgcgttgtgccgcttcaatgcgggcgcgtccgctggaaaagtcctgctcaatgtactttttcggcttctgtgatccggtcatcgttcgagcaatctccattaggtcggccagccgatccacacgatcatgctggcagtgccatttataggctgtcggatcgtctgagacgtgcagcggccaccggctcagcctatgcgaaaaagcctggtcagcgccgaaaacacgagtcatttcttccgtcgttgcagccagcaggcgcatatttgggctggttttacctgctgcggcatacaccgggtcaatgagccagatgagctggcatttcccgctcagcggattcacgccgatccaagccggcgctttttctaggcgtgcccatttctctaaaatcgcgtagacctgcgggtttacgtgctcaatcttcccgccggcctggtggctgggcacatcgatgtcaagcacgatcaccgcggcatgttgcgcgtgcgtcagcgcaacgtactggcaccgcgtcagcgcttttgagccagcccggtagagctttggttgggtttcgccggtatccgggtttttaatccaggcgctcgcgaaatctcttgtcttgctgccctggaagctttcgcgtcccaggtgagcgagcagttcgcggcgatcttctgccgtccagccgcgtgagccgcagcgcatagcttcggggtgggtgtcgaacagatcggcggacaatttccacgcgctagctgtgactgtgtcctgcggatcggctagagtcatgtcttgagtgctttctcccagctgatgactgggggttagccgacgccctgtgagttcccgctcacggggcgttcaactttttcaggtatttgtgcagcttatcgtgttttcttcgtaaatgaacgcttaactaccttgttaaacgtggcaaataggcaggattgatggggatctagcttcacgctgccgcaagcactcagggcgcaagggctgctaaaggaagcggaacacgtagaaagccagtccgcagaaacggtgctgaccccggatgaatgtcagctactgggctatctggacaagggaaaacgcaagcgcaaagagaaagcaggtagcttgcagtgggcttacatggcgatagctagactgggcggttttatggacagcaagcgaaccggaattgccagctggggcgccctctggtaaggttgggaagccctgcaaagtaaactggatggctttcttgccgccaaggatctgatggcgcaggggatcaagatctgatcaagagacaggatgaggatcgtttcgcatgattgaacaagatggattgcacgcaggttctccggccgcttgggtggagaggctattcggctatgactgggcacaacagacaatcggctgctctgatgccgccgtgttccggctgtcagcgcaggggcgcccggttctttttgtcaagaccgacctgtccggtgccctgaatgaactccaagacgaggcagcgcggctatcgtggctggccacgacgggcgttccttgcgcagctgtgctcgacgttgtcactgaagcgggaagggactggctgctattgggcgaagtgccggggcaggatctcctgtcatctcaccttgctcctgccgagaaagtatccatcatggctgatgcaatgcggcggctgcatacgcttgatccggctacctgcccattcgaccaccaagcgaaacatcgcatcgagcgagcacgtactcggatggaagccggtcttgtcgatcaggatgatctggacgaagagcatcaggggctcgcgccagccgaactgttcgccaggctcaaggcgcggatgcccgacggcgaggatctcgtcgtgacccatggcgatgcctgcttgccgaatatcatggtggaaaatggccgcttttctggattcatcgactgtggccggctgggtgtggcggaccgctatcaggacatagcgttggctacccgtgatattgctgaagagcttggcggcgaatgggctgaccgcttcctcgtgctttacggtatcgccgctcccgattcgcagcgcatcgccttctatcgccttcttgacgagttcttctgagcgggactctggggttcgcggaatcatgaccaaaatcccttaacgtgagttttcgttccactgagcgtcagaccccgtagaaaagatcaaaggatcttcttgagatcctttttttctgcgcgtaatctgctgcttgcaaacaaaaaaaccaccgctaccagcggtggtttgtttgccggatcaagagctaccaactctttttccgaaggtaactggcttcagcagagcgcagataccaaatactgtccttctagtgtagccgtagttaggccaccacttcaagaactctgtagcaccgcctacatacctcgctctgctaatcctgttaccagtggctgctgccagtggcgataagtcgtgtcttaccgggttggactcaagacgatagttaccggataaggcgcagcggtcgggctgaacggggggttcgtgcacacagcccagcttggagcgaacgacctacaccgaactgagatacctacagcgtgagctatgagaaagcgccacgcttcccgaagggagaaaggcggacaggtatccggtaagcggcagggtcggaacaggagagcgcacgagggagcttccagggggaaacgcctggtatctttatagtcctgtcgggtttcgccacctctgacttgagcgtcgatttttgtgatgctcgtcaggggggcggagcctatggaaaaacgccagcaacgcggcctttttacggttcctggccttttgctggccttttgctcacatgttctttcctgcgttatcccctgattctgtggataaccgtattaccgcctttgagtgagctgataccgctcgccgcagccgaacgaccgagcgcagcgagtcagtgagcgaggaagcggaagagcgcctgatgcggtattttctccttacgcatctgtgcggtatttcacaccgcatatggtgcactctcagtacaatctgctctgatgccgcatagttaagccagtatacactccgctatcgctacgtgactgggtcatggctgcgccccgacacccgccaacacccgctgacgcgccctgacgggcttgtctgctcccggcatccgcttacagacaagctgtgaccgtctccgggagctgcatgtgtcagaggttttcaccgtcatcaccgaaacgcgcgaggcagcagatcaattcgcgcgcgaaggcgaagcggcatgcatttacgttgacaccatcgaatggtgcaaaacctttcgcggtatggcatgatagcgcccggaagagagtcaattcagggtggtgaatgtgaaaccagtaacgttatacgatgtcgcagagtatgccggtgtctcttatcagaccgtttcccgcgtggtgaaccaggccagccacgtttctgcgaaaacgcgggaaaaagtggaagcggcgatggcggagctgaattacattcccaaccgcgtggcacaacaactggcgggcaaacagtcgttgctgattggcgttgccacctccagtctggccctgcacgcgccgtcgcaaattgtcgcggcgattaaatctcgcgccgatcaactgggtgccagcgtggtggtgtcgatggtagaacgaagcggcgtcgaagcctgtaaagcggcggtgcacaatcttctcgcgcaacgcgtcagtgggctgatcattaactatccgctggatgaccaggatgccattgctgtggaagctgcctgcactaatgttccggcgttatttcttgatgtctctgaccagacacccatcaacagtattattttctcccatgaagacggtacgcgactgggcgtggagcatctggtcgcattgggtcaccagcaaatcgcgctgttagcgggcccattaagttctgtctcggcgcgtctgcgtctggctggctggcataaatatctcactcgcaatcaaattcagccgatagcggaacgggaaggcgactggagtgccatgtccggttttcaacaaaccatgcaaatgctgaatgagggcatcgttcccactgcgatgctggttgccaacgatcagatggcgctgggcgcaatgcgcgccattaccgagtccgggctgcgcgttggtgcggatatctcggtagtgggatacgacgataccgaagacagctcatgttatatcccgccgtcaaccaccatcaaacaggattttcgcctgctggggcaaaccagcgtggaccgcttgctgcaactctctcagggccaggcggtgaagggcaatcagctgttgcccgtctcactggtgaaaagaaaaaccaccctggcgcccaatacgcaaaccgcctctccccgcgcgttggccgattcattaatgcagctggcacgacaggtttcccgactggaaagcgggcagtgagcgcaacgcaattaatgtgagttagcgcgaattgatctggtttgacagcttatcatcgactgcacggtgcaccaatgcttctggcgtcaggcagccatcggaagctgtggtatggctgtgcaggtcgtaaatcactgcataattcgtgtcgctcaaggcgcactcccgttctggataatgttttttgcgccgacatcataacggttctggcaaatattctgaaatgagctgttgacaattaatcatccggctcgtataatgtgtggaattgtgagcggataacaatttcacacaggaaacagtgaacgttaaaaaggaatttcgtatgagcattctggatttcccgcgtatccacttccgtggctgggcccgtgtcaatgcgccgaccgcgaaccgcgatccgcacggccacatcgatatggccagcaataccgtggcgatggcgggtgagccgttcgacctggcacgccatcctacggagttccaccgtcacctgcgctccctgggtccgcgcttcggcttggatggtcgtgctgacccggaaggcccgttcagcctggccgagggctacaacgctgccggtaacaaccacttttcgtgggagagcgcaaccgttagccacgtgcaatgggatggcggtgaggcggatcgtggtgacggtctggtcggtgctcgtttggcactgtggggtcactacaatgattatctgcgtaccaccttcaatcgtgctcgttgggtcgacagcgacccgacgcgccgtgacgctgcacaaatctatgcgggccaattcaccattagcccggctggtgccggtccgggtacgccgtggctgtttacggcagacattgatgatagccatggtgcacgttggacgcgtggcggccacattgcagagcgtggcggccacttcttggatgaagagtttggtctggcacgcctgtttcagttctctgtgccgaaagatcacccacattttctgtttcacccgggtccgtttgattccgaggcctggcgtcgtctgcaattggctctggaggatgacgacgttctgggtctgaccgtgcaatatgcgttgttcaatatgagcaccccgcctcagccgaacagcccggtttttcacgatatggtcggtgttgtcggtctgtggcgtcgtggtgaactggcgagctacccggctggtcgtctgctgcgtccgcgtcaaccgggtctgggtgacctgaccctgcgcgtcaacggtggtcgcgttgcgctgaatttggcgtgtgccattccgttcagcactcgtgccgcgcagccaagcgcaccggaccgcctgaccccggacctgggtgccaaactgccgctgggcgatctgctgctgcgtgatgaggacggcgcactgttggcacgtgtgccgcaggctctgtaccaagactattggacgaatcacggtattgtggacctgccgctgctgcgcgaaccgcgtggtagcttgaccctgagcagcgaactggcggagtggcgtgagcaagactgggtcacccaaagcgacgcgtctaacctgtacctggaggcaccggatcgccgtcacggtcgctttttccctgagagcatcgcgctgcgcagctactttcgcggtgaagcgcgtgcgcgtccggatatcccgcatcgtatcgagggcatgggcctggtcggcgtcgaatctcgtcaggatggcgacgctgcggaatggcgtctgacgggtctgcgtccgggtccggcacgcattgttctggacgatggtgccgaggcgatccctctgcgtgttctgcctgacgattgggcgctggatgacgcgaccgtcgaagaagtggattacgcctttttgtaccgccacgttatggcgtattacgagctggtgtatccattcatgagcgacaaggtgttttccctggctgatcgttgcaaatgtgaaacgtacgcacgtctgatgtggcagatgtgtgatccgcagaaccgcaacaagtcctattacatgccgagcacccgcgaactgtcggcaccgaaagctcgtttgttcttgaagtatctggcccacgtggaaggccaggcacgcctgcaagcacctccgccagcgggtccggcacgcattgaatctaaagcccagttggcggcagagctgcgtaaagccgtcgacctggagctgtctgtgatgctgcaatacctgtacgcggcgtatagcattccgaactatgcacagggccaacaacgtgttcgtgacggtgcgtggaccgccgagcagctgcaactggcgtgcggtagcggtgaccgtcgccgtgatggcggtattcgtgcagcactgctggaaattgctcatgaagaaatgattcattacctggtcgttaacaacctgctgatggccctgggcgagccgttctacgcgggtgtcccgctgatgggcgaagcggcacgtcaggcgtttggcctggacaccgagttcgctctggaaccgtttagcgaaagcacgctggcacgttttgttcgtctggaatggccgcactttatcccagcaccgggcaaatccatcgcggactgctatgccgccattcgtcaggcgtttttggatctgccggacttgtttggtggcgaggcaggtaagcgtggcggtgaacaccacctgttcctgaatgagctgaccaaccgtgcgcatccgggttatcaactggaagttttcgatcgcgactcggcgctgtttggtattgcatttgtgaccgatcagggcgaaggtggcgctctggacagcccgcactacgaacatagccattttcaacgtctgcgtgaaatgagcgcgcgtatcatggctcaaagcgcaccgttcgaaccggcgctgccggcgttgcgtaatccggttctggatgagagcccgggttgccaacgtgtcgcagacggtcgtgcgcgtgcgctgatggcattgtaccaaggcgtttatgagctgatgtttgcgatgatggcgcagcacttcgccgtgaaaccgctgggtagcttgcgtcgcagccgcctgatgaacgcagcaatcgatctgatgaccggtctgttgcgtccgctgagctgcgcgctgatgaacctgccaagcggcatcgccggtcgcacggccggtccgccgctgccgggtccggttgacacccgtagctatgacgactacgcgctgggctgtcgcatgctggcacgccgttgcgagcgtctgctggagcaggcgagcatgctggaaccgggttggctgccggatgcgcagatggagctgctggatttctatcgtcgccaaatgctggacttggcgtgcggcaaactgagccgcgaggcctaataggaaaggaggtttggacaatgaagcactcttctgacatctgtatcgttggtgctggtatctctggtttgacctgtgcttctcacttgttggactctccagcttgtagaggtttgtctttgagaatcttcgacatgcaacaagaagctggtggtagaatccgatctaagatgttggacggtaaggcttctatcgaattgggtgctggtagatactctccacaattgcacccacacttccaatctgctatgcaacactactctcaaaagtctgaagtttacccattcacccaattgaagttcaagtctcacgttcaacaaaagttgaagagagctatgaacgaattgtctccaagattgaaggaacacggtaaggaatctttcttgcaattcgttagtagataccaaggtcacgactctgctgttggtatgatccgatctatgggttacgacgctttgttcttgccagacatctctgctgaaatggcttacgacatcgttggtaagcacccagaaatccaatctgttaccgacaacgacgctaaccaatggttcgctgctgaaacaggtttcgctggtttgatccaaggtatcaaggctaaggttaaggctgctggtgctagattctctttgggttacagattgttgtctgttagaaccgacggtgacggttacttgttgcaattggctggtgacgacggttggaagttggaacacagaaccagacacttgatcttggctatcccaccatctgctatggctggtttgaacgttgacttcccagaggcttggtctggtgctagatacggttctttgccattgttcaagggtttcttgacctacggtgaaccatggtggttggactacaagttggacgaccaagttttgatcgttgacaacccattgagaaaaatctacttcaagggtgacaagtacttgttcttctacaccgactctgaaatggctaactactggagaggttgtgttgctgaaggtgaagacggttacttggaacaaatcagaacccacttggcttctgctttgggtatcgttagagaaagaatcccacaaccattggctcacgttcacaagtactgggctcacggtgttgagttctgtagagactctgacatcgaccacccatctgctttgtctcacagagactctggtatcatcgcttgttctgacgcttacaccgaacactgtggttggatggaaggtggtttgttgtctgctagagaggctagtagattgttgttgcaaagaatcgctgctttgagaagataggaaaggaggtttggacaatggaaaacagagaaccaccattgttgccagctagatggtcttctgcttacgtttcttactggagtccaatgttgccagacgaccaattgacctctggttactgttggttcgactacgaaagagacatctgtagaatcgacggtttgttcaacccgtggtctgaaagagacaccggttacagattgtggatgtctgaagttggtaacgctgcttctggtagaacctggaagcaaaaggttgcttacggtagagaaagaaccgctttgggtgaacaattgtgtgaaagaccattggacgacgaaaccggtccattcgctgaattgttcttgccacgcgacgttttgagaagattgggtgctagacacatcggtagaagagttgttttgggtagagaagctgacggttggagataccaaagaccaggtaagggtccatctaccttgtacttggacgctgcttctggtaccccattgagaatggttaccggtgacgaggctagtagagcttctttgagagacttcccaaacgtttctgaagctgaaatcccagacgctgttttcgctgctaagagataggaaaggaggtttggacaatgaaaagagctatcatcgttggtggtggtttggctggtggtttgaccgctatctacttggctaagagaggttacgaagttcacgttgttgaaaagagaggtgacccattgagagacttgtcttcttacgttgacgttgtttctagtagagctatcggtgtttctatgaccgttagaggtatcaagtctgttttggctgctggtatcccaagagctgaattggacgcttgtggtgaaccaatcgttgctatggctttctctgttggtggtcaatacagaatgagagaattgaagccattggaagacttcagaccattgtctttgaacagagctgctttccaaaagttgttgaacaagtacgctaacttggctggtgttagatactacttcgaacacaagtgtttggacgttgacttggacggtaagtctgttttgatccaaggtaaggacggtcaaccacaaagattgcaaggtgacatgatcatcggtgctgacggtgctcactctgctgttagacaagctatgcaatctggtttgagaagattcgagttccaacaaaccttcttcagacacggttacaagaccttggttttgccagacgctcaggctttgggttacagaaaggacaccttgtacttcttcggtatggactctggtggtttgttcgctggtagagctgctaccatcccagacggttctgtttctatcgctgtttgtttgccatactctggttctccatctttgaccaccaccgacgaaccaaccatgagagctttcttcgacagatacttcggtggtttgccacgcgacgctcgcgacgaaatgttgagacaattcttggctaagccatctaacgacttgatcaacgttcgatcttctaccttccactacaagggtaacgttttgttgttgggtgacgctgctcacgctaccgctccattcttgggtcaaggtatgaacatggctttggaagacgctagaaccttcgttgaattgttggacagacaccaaggtgaccaagacaaggctttcccagagttcaccgaattgagaaaggttcaagctgacgctatgcaagacatggctagagctaactacgacgttttgtcttgttctaacccaatcttcttcatgagagctagatacaccagatacatgcactctaagttcccaggtttgtacccaccagacatggctgaaaagttgtacttcacctctgaaccatacgacagattgcaacaaatccaaagaaagcaaaacgtttggtacaagatcggtagagttaactaggaaaggaggtttggacaatgaaaatcttggttatcggtgctggtccagctggtttggttttcgcttctcaattgaagcaagctagaccattgtgggctatcgacatcgttgaaaagaacgacgaacaagaagttttgggttggggtgttgttttgccaggtagaccaggtcaacacccagctaacccattgtcttacttggacgctccagaaagattgaacccacaattcttggaagacttcaagttggttcaccacaacgaaccatctttgatgtctaccggtgttttgttgtgtggtgttgaaagaagaggtttggttcacgctttgagagacaagtgtcgatctcaaggtatcgctatcagattcgaatctccattgttggaacacggtgaattgccattggctgactacgacttggttgttttggctaacggtgttaaccacaagaccgctcacttcaccgaggctttggttccacaagttgactacggtagaaacaagtacatctggtacggaacctctcaattgttcgaccaaatgaacttggttttcagaacccacggtaaggacatcttcatcgctcacgcttacaagtactctgacaccatgtctaccttcatcgttgaatgttctgaagaaacctacgctagagctagattgggtgaaatgtctgaagaggcttctgctgaatacgttgctaaggttttccaagctgaattgggtggtcacggtttggtttctcaaccaggtttgggttggagaaacttcatgaccttgtctcacgacagatgtcacgacggtaagttggttttgttgggtgacgctttgcaatctggtcacttctctatcggtcacggaaccacaatggctgttgttgttgctcaattgttggttaaggctttgtgtaccgaagacggtgttccagctgctttgaagagattcgaagaaagagctttgccattggttcaattgttcagaggtcacgctgacaacagtagagtttggttcgaaaccgttgaagaaagaatgcacttgtcttctgctgagttcgttcaatctttcgacgctagaagaaagtctttgccaccaatgccagaggctttggctcaaaacttgagatacgctttgcaaagatagccggataaaacgaaaggctcagtcgaaagactgggcctttcgttttatctgttgtttgtcggtgaacgctctcctgagtaggacaaatccgccg
